# Supplementary material for: Computational analysis of peripheral blood smears detects disease-associated cytomorphologies
Source: Nat Commun. 2023 Jul 20;14:4378. doi: 10.1038/s41467-023-39676-y (PMC10359268; doi:10.1038/s41467-023-39676-y)
Supplement: Supplementary file 1 — Supplementary Information [file 41467_2023_39676_MOESM1_ESM.pdf]

Computational analysis of peripheral blood smears detects disease-associated cytomorphologies

José Guilherme de Almeida[1,2], Emma Gudgin[3], Martin Besser[3], William G. Dunn[3], Jonathan Cooper[4], Torsten Haferlach[5], George S. Vassiliou[4,6,7,*], and Moritz Gerstung[1,8,*]

[1]European Bioinformatics Institute — EMBL-EBI, Hinxton, CB10 1SD, UK
[2]Currently: Champalimaud Foundation—Centre for the Unknown, 1400-038 Lisbon, Portugal
[3]Department of Haematology, Cambridge Institute for Medical Research, University of Cambridge, Cambridge, UK
[4]Wellcome Sanger Institute, Wellcome Genome Campus, Cambridge, CB10 1SD, UK
[5]Munich Leukemia Laboratory GmbH, Munich, Germany
[6]Wellcome-MRC Cambridge Stem Cell Institute, University of Cambridge, Cambridge, CB2 0XY, UK
[7]Department of Haematology, University of Cambridge, Cambridge, CB2 0XY, UK
[8]Division for AI in Oncology, German Cancer Research Center (DKFZ), Im Neuenheimer Feld 280, 69120 Heidelberg, Germany
* Correspondence to G.S.V, gsv20@cam.ac.uk or M.G., moritz.gerstung@dkfz.de

Supplementary Information

Supplementary Figures

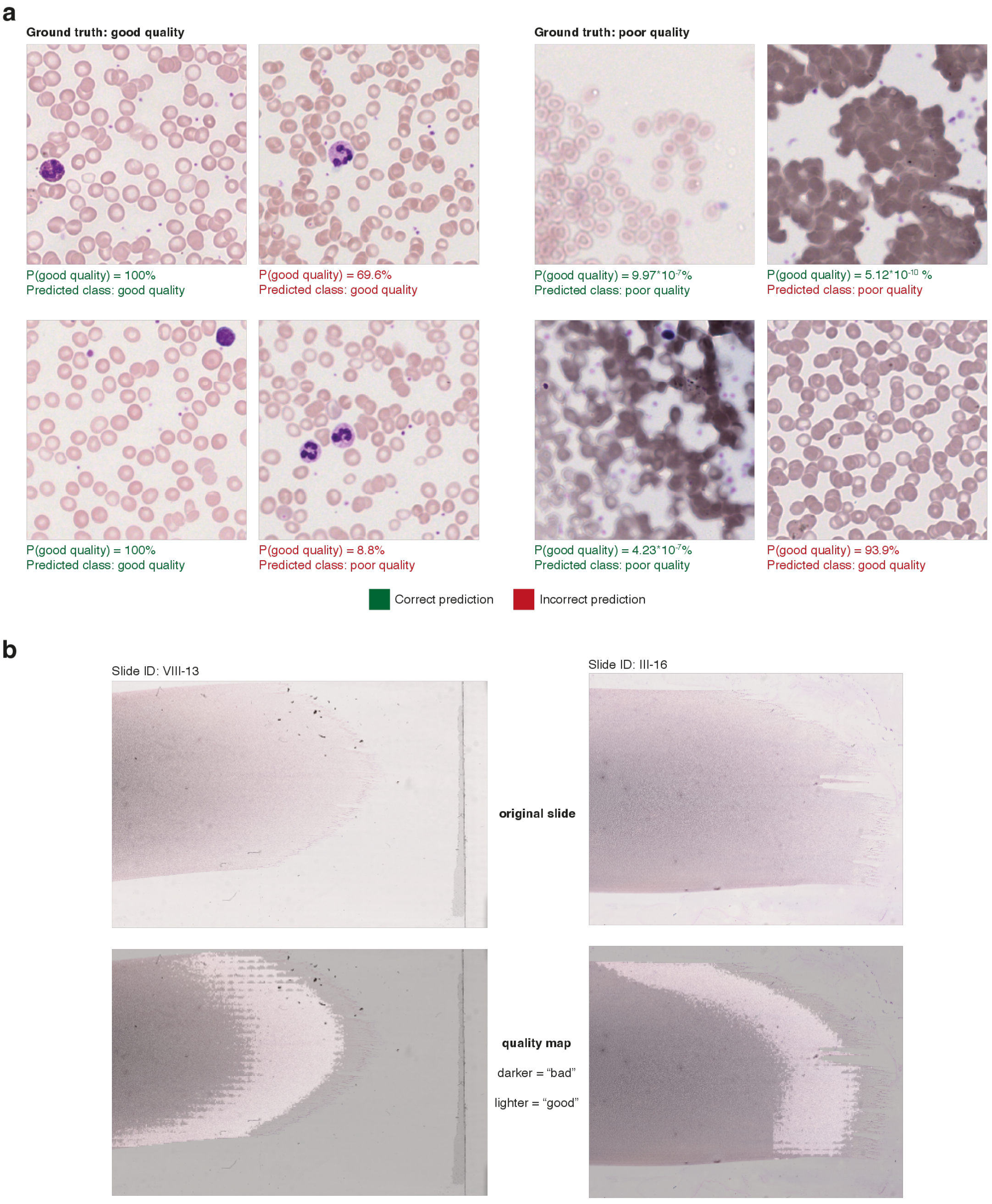

Supplementary Figure S1 - **Quality control examples. a** — Good and poor quality tiles and their respective predictions and prediction probability according to a neural network trained to predict tile quality. **b** — Quality maps (regions of the slide classified as being of good quality) for two PBS. Examples were collected randomly for both **a** and **b**.

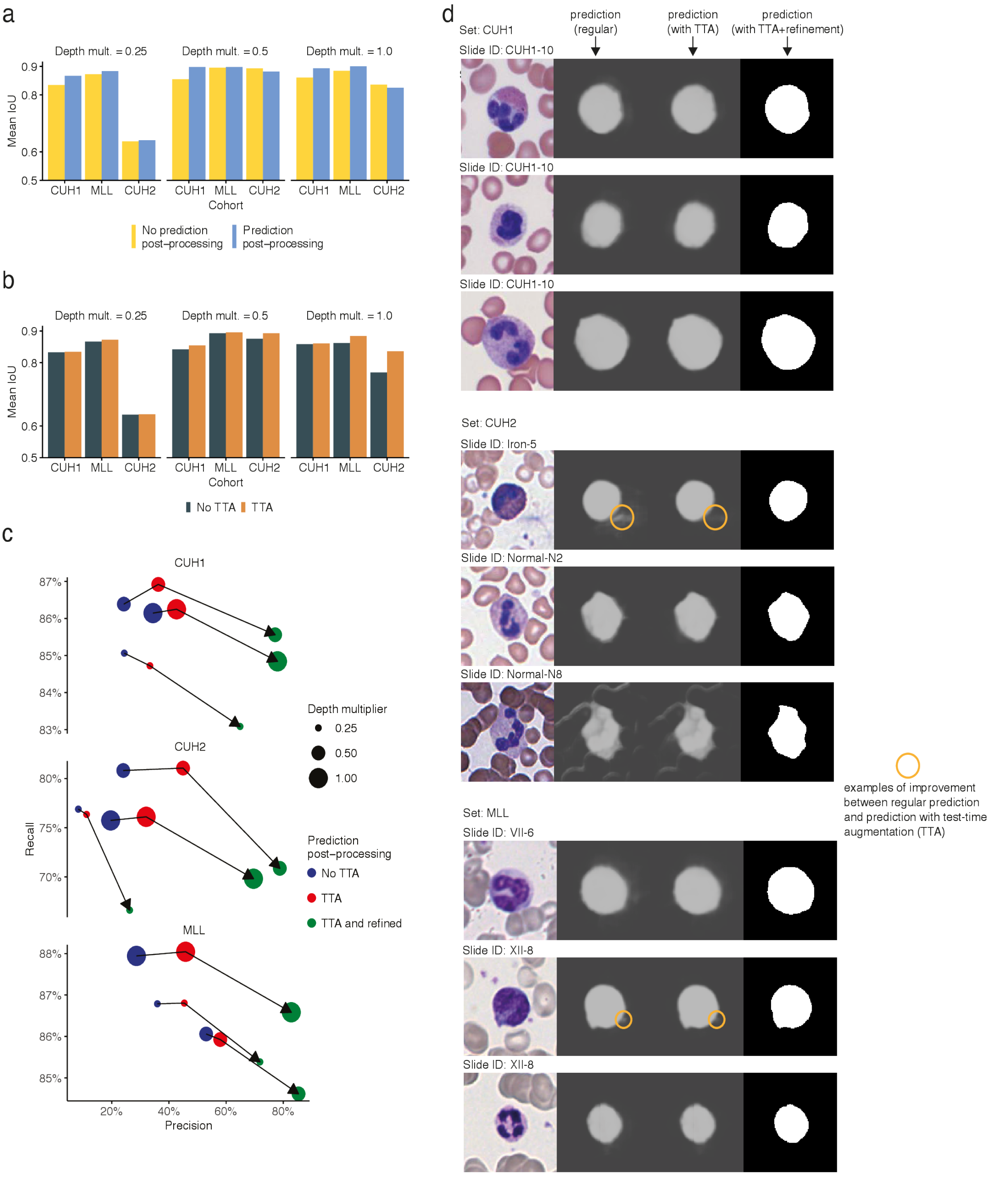

Supplementary Figure S2 - **U-Net WBC segmentation performance. a** — Neural network performance with and without prediction post-processing stratified by network depth and dataset. **b** — Neural network performance with and without test-time augmentation (TTA) stratified by network depth and dataset. **c** – Object detection metrics with TTA and with prediction post-processing (refine). The arrow direction symbolizes the addition of extra steps (sequentially: no TTA, TTA, TTA and refine). **d** — Randomly extracted WBC segmentation examples. For both **a** and **b**, the training dataset is always CUH1, whereas the other dataset (the MLL dataset and CUH2) are validation dataset.

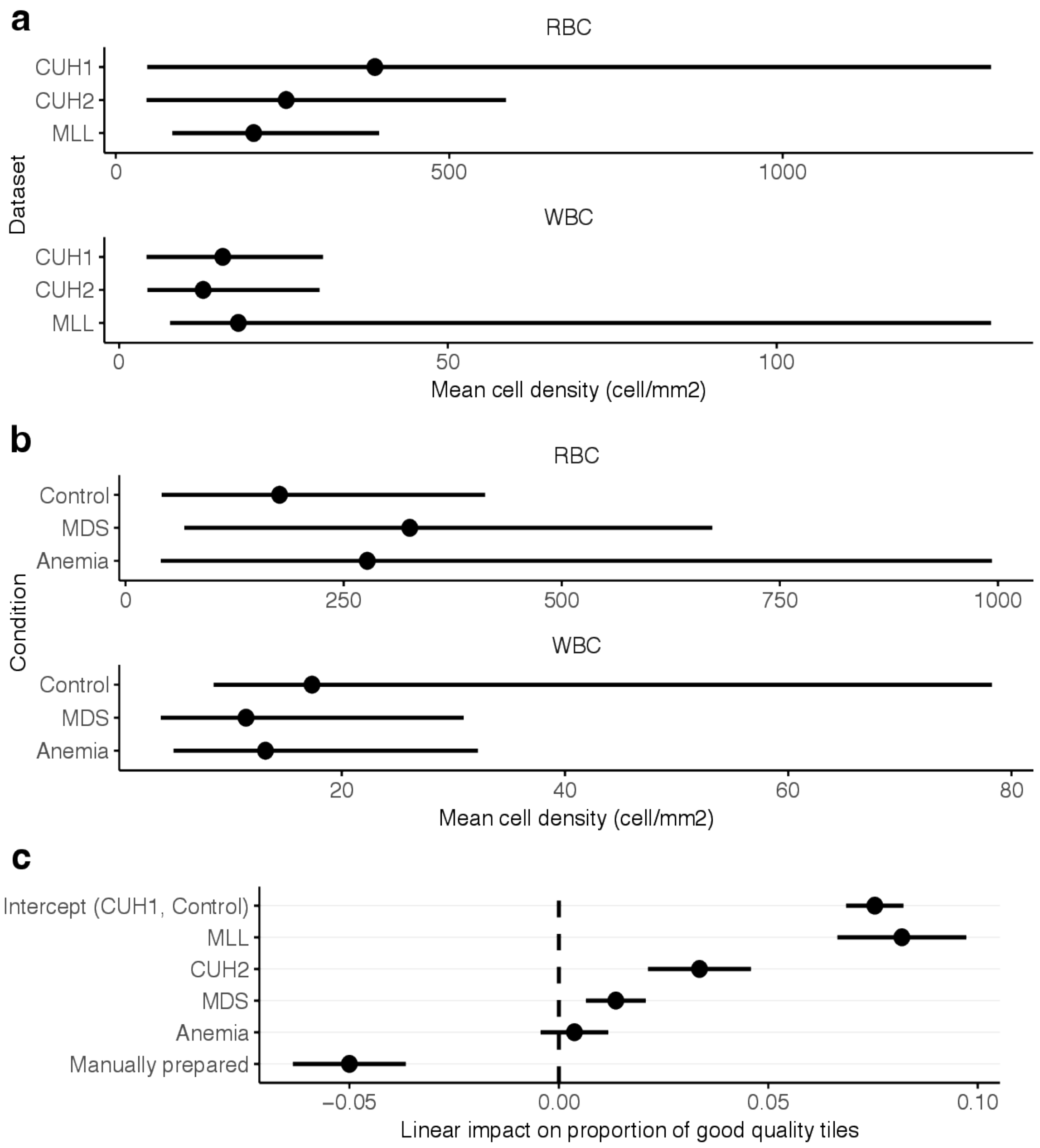

Supplementary Figure S3 - **Impact of cohort, condition and slide preparation on cellular density and tile quality. a** — Distribution of cellular density for RBC and WBC stratified by cohort (n=54, 68 and 353 independent PBS for CUH1, CUH2 and MLL, respectively). **b** —Distribution of cellular density for RBC and WBC stratified by condition (n=122, 261, 92 independent PBS for controls, MDS and anaemia, respectively). **c** – Linear impact of different factors on the proportion of good quality tiles per slide. In **a** and **b**, the circles represent the median, whereas the leftmost and rightmost ends of the horizontal lines correspond to the 5% and 95% quantiles, respectively. In **c**, the circles and lines are the coefficient estimates and standard error, respectively, for a linear model where the proportion of good quality tiles is the dependent variable and the condition (Control vs. MDS vs. Anemia), cohort (CUH1 vs. CUH2 vs. MLL) and the preparation (Manually prepared vs. Automatically prepared) are the independent variables. The Intercept corresponds to the proportion of good tiles in automatically prepared CUH1 controls.

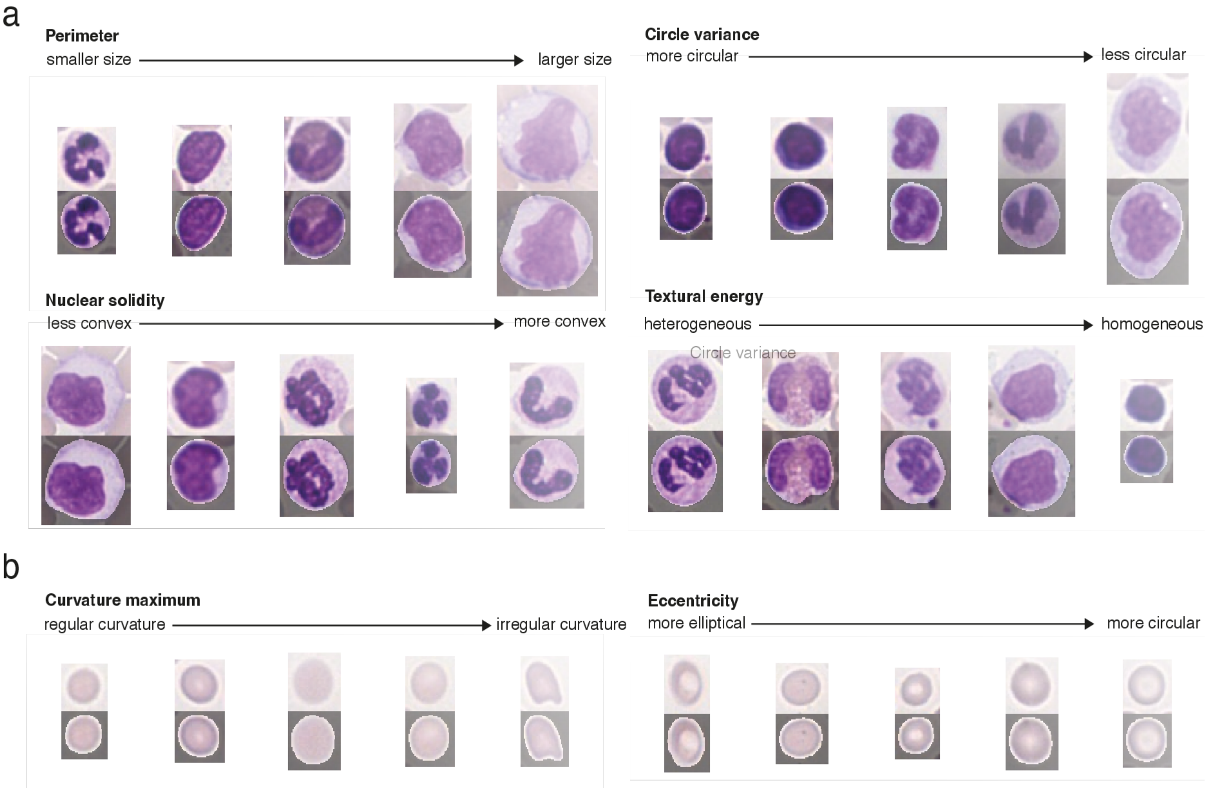

Supplementary Figure S4 - **Examples of cellular characterization using the protocol delineated in this work. a** — WBC examples. **b** — RBC examples.

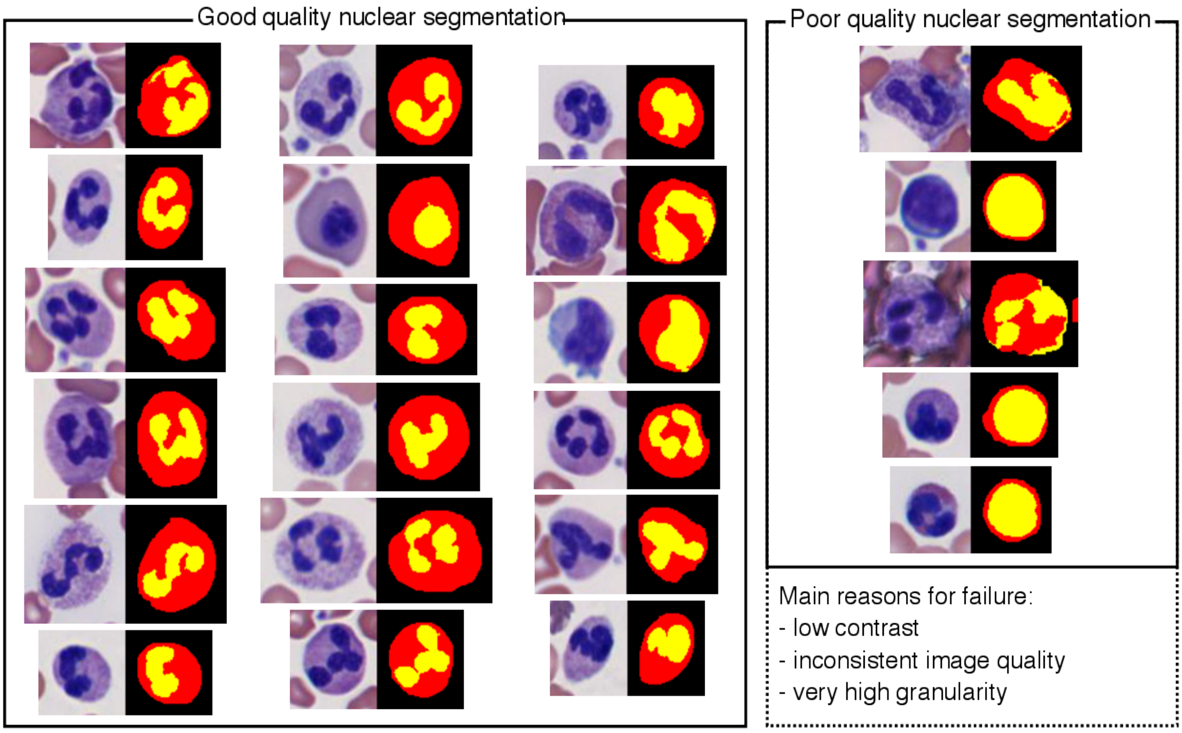

Supplementary Figure S5 - **Examples of WBC nuclear segmentation and failure cases (poor quality nuclear segmentation).** Red represents cytoplasm and yellow represents the nucleus. Failure cases are observable in situations where image contrast is relatively low, granularity is high or the image is of relatively poor quality. These examples were randomly collected across all slides and subsequently categorized under good or poor quality nuclear segmentation.

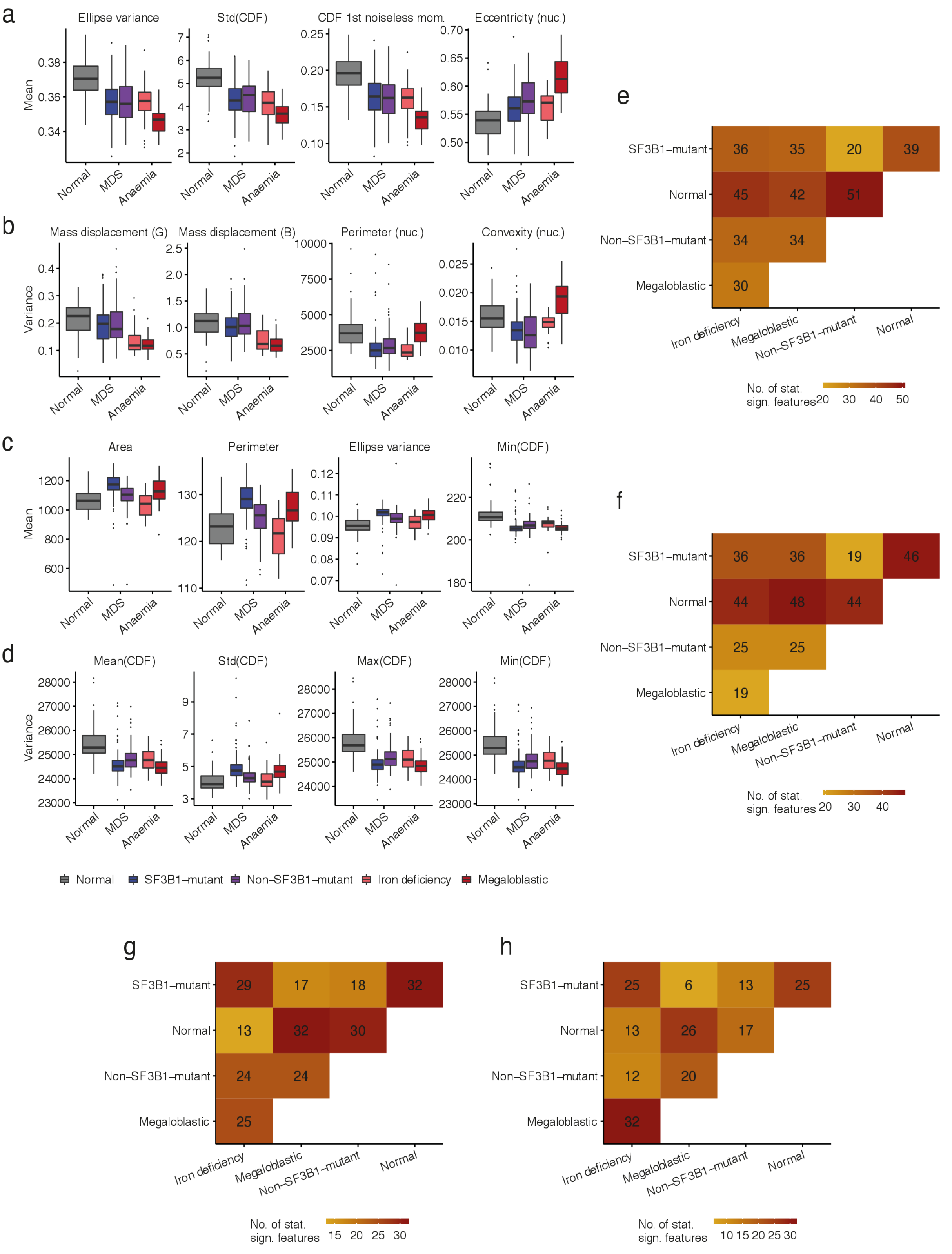

Supplementary Figure S6 - **Individual aspects of morphometric feature distribution carry discriminatory power to predict different conditions. a-d** — Distribution of 4 highly discriminatory feature means in WBC, feature variances in WBC, feature means in RBC and feature variances in RBC, respectively. Highly discriminating features were detected as having the highest Kruskal-Wallis statistic out of all features (n=57, 222 and 65 independent PBS for controls (Normal), MDS and Anemia, respectively; each boxplot represents the extrema (tips of whiskers), 25% and 75% quantiles (upper and lower edges of boxes, respectively), median (center of box) and outliers (black points not contained within 1.5 interquantile ranges of the 25% and 75% quantiles)). **e-h** — Number of highly discriminatory WBC feature means, WBC feature variances, RBC feature means

and RBC feature variances, respectively, for all condition combinations (according to significant comparisons in a Dunn-Bonferroni test on features which were significant for a Kruskal-Wallis test).

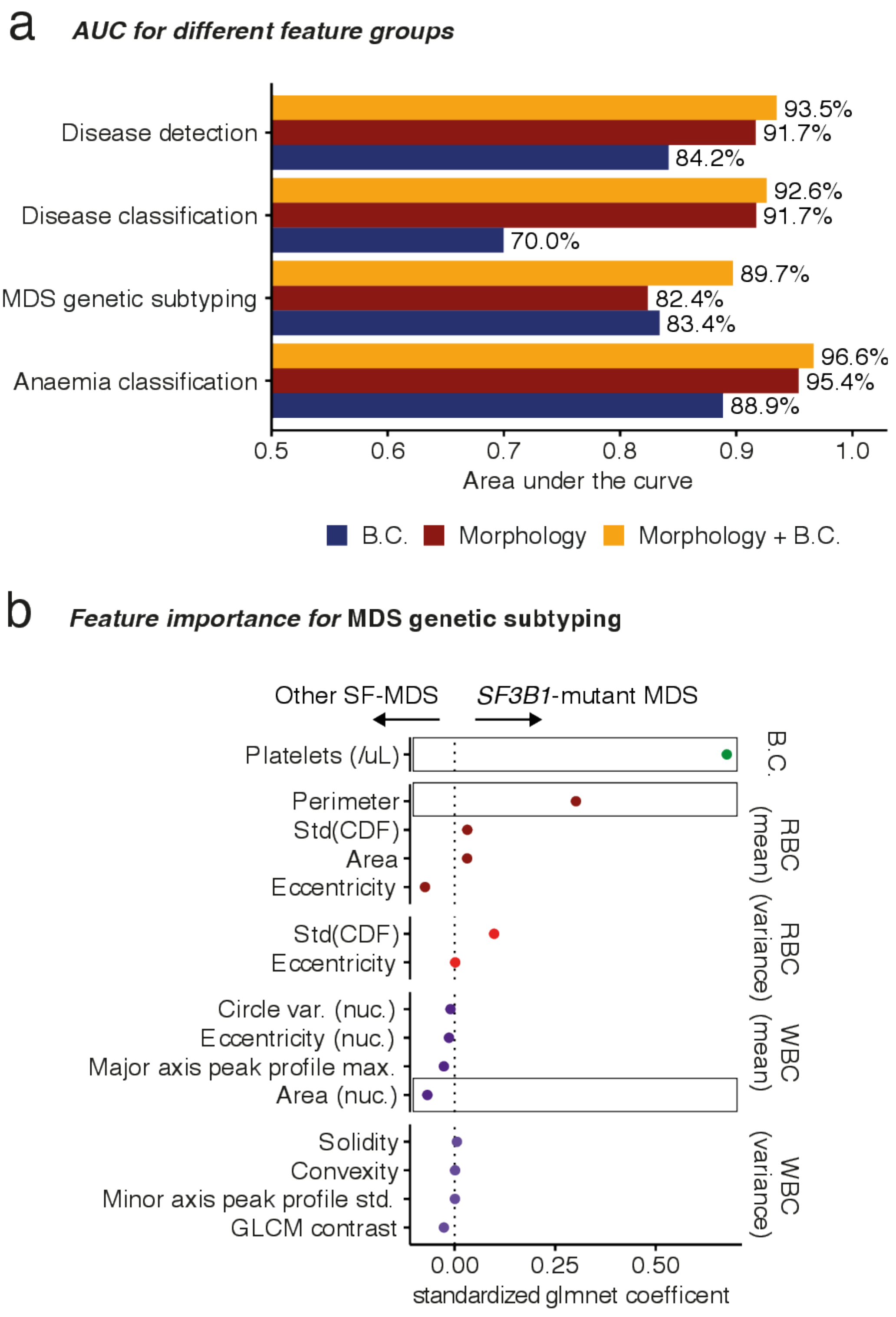

Supplementary Figure S7 - **Automated morphology improves condition prediction. a** — Cross-validated area under the ROC (AUC) curve. In **a**, lines and bars are coloured according to the used datasets for each task. **b** — Individual feature importance (i.e. coefficients for standardized features) for MDS genetic subtyping using glmnet.

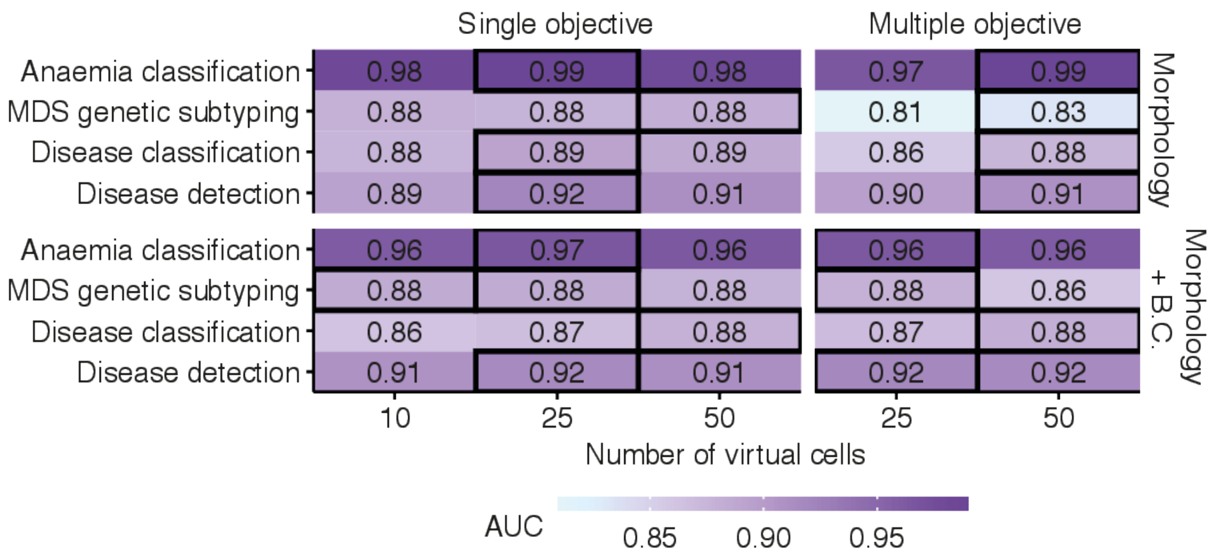

Supplementary Figure S8 - **Performance (AUC) of the multiple and single objective Morphotype analysis model according to the assumed number of computational morphotypes and for different tasks.**

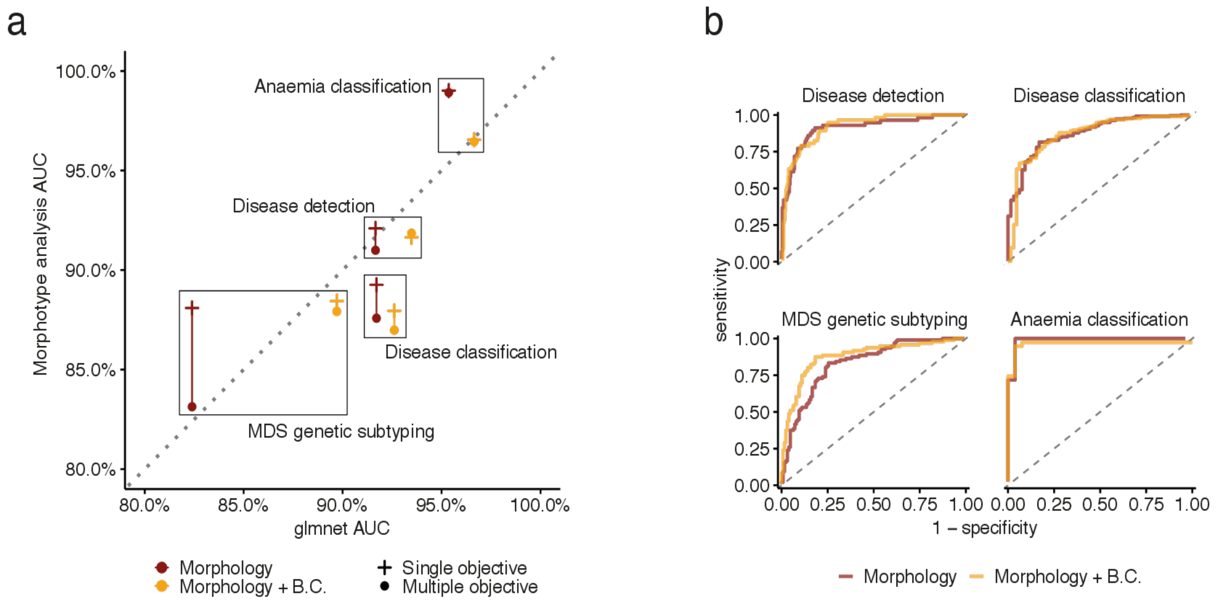

Supplementary Figure S9 - **Predictive performance of morphotype analysis and comparison with glmnet. a** - Comparison of the cross-validated AUC for glmnet and single and multiple objective Morphotype analysis. **b** - Receiver-operating characteristic curves for the predictive performance of Morphotype analysis across different tasks.

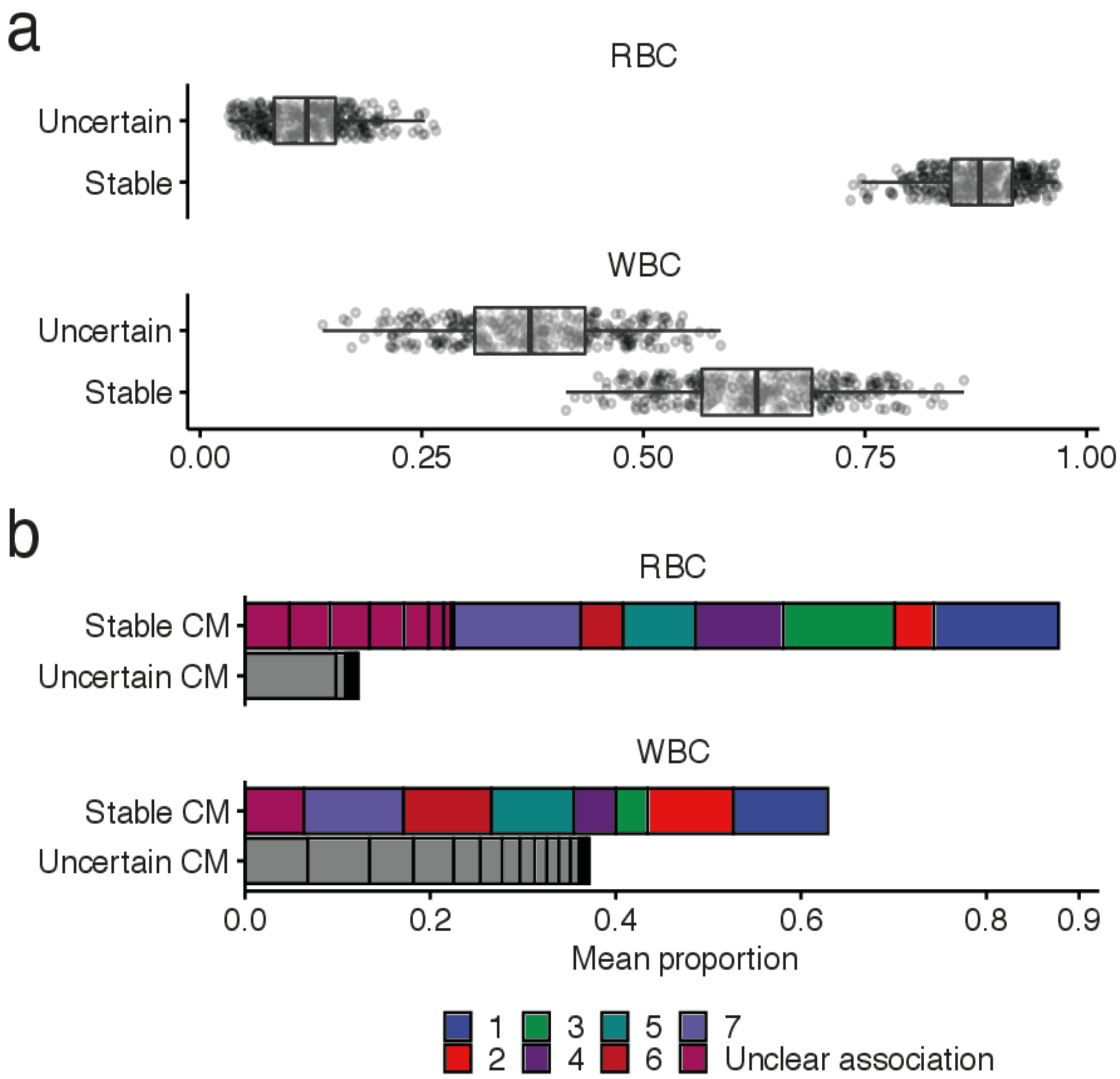

Supplementary Figure S10 - **Morphotype stability in WBC in RBC. a** - Stable and uncertain RCMs (top) and WCMs (bottom) per slide (n=353 independent PBS; each boxplot represents the extrema (tips of whiskers), 25% and 75% quantiles (upper and lower edges of boxes, respectively) and median (center of box)). **b** - Mean proportion of stable RCMs (top) and WCMs (bottom) stratified by CM identity. Colours correspond to different RCMs/WCMs and the bar fractions corresponding to CMs with unclear associations/uncertain CMs are divided by black lines according to the mean prevalence of their constituting CMs.

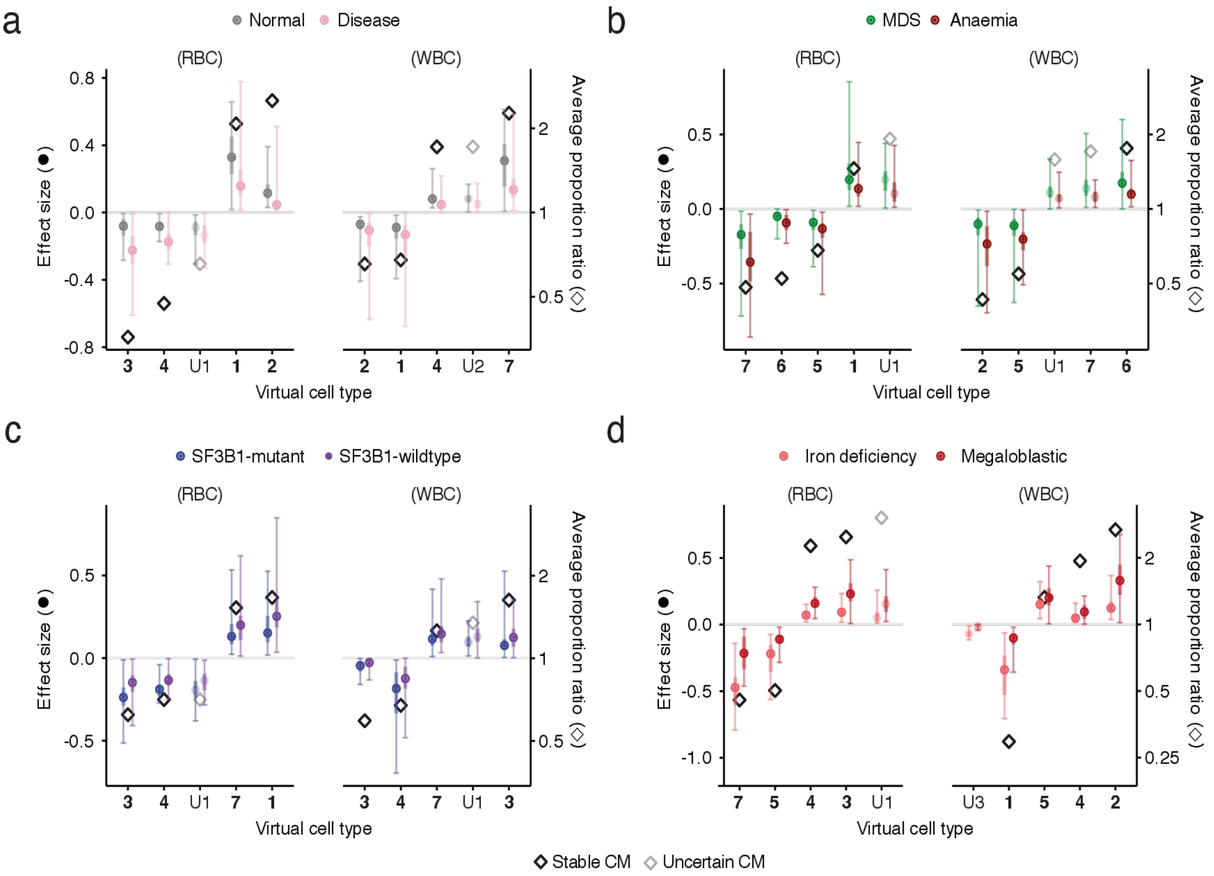

Supplementary Figure S11 - **Effect sizes for the most discriminative computational morphotype (CM) proportions for the multiple objective Morphotype analysis predicting different tasks. a** — Disease detection (normal vs. disease). Effect sizes above zero are biassed towards the "normal" classification. **b** — Disease classification (MDS vs. anemia). Effect sizes above zero are biassed towards the "MDS" classification. **c** — *SF3B1*-mutant detection (*SF3B1*-mutant MDS vs. *SF3B1*-wildtype MDS). Effect sizes above zero are biassed towards the *SF3B1*-wildtype MDS classification. **d** — anemia classification (iron deficiency vs. megaloblastic). Effect sizes above zero are biassed towards the "megaloblastic" classification. For **a-d**, the effect size was calculated as the product of the proportion of each CM and its coefficient in Morphotype analysis. Each point (circle) represents the median effect size and the error bars represent the 90% confidence interval for the effect sizes and corresponds to the left y-axis. Each diamond represents the proportion ratio for each condition and corresponds to the right y-axis (n=9,091,220 RBC and 434,436 WBC across 353 PBS).

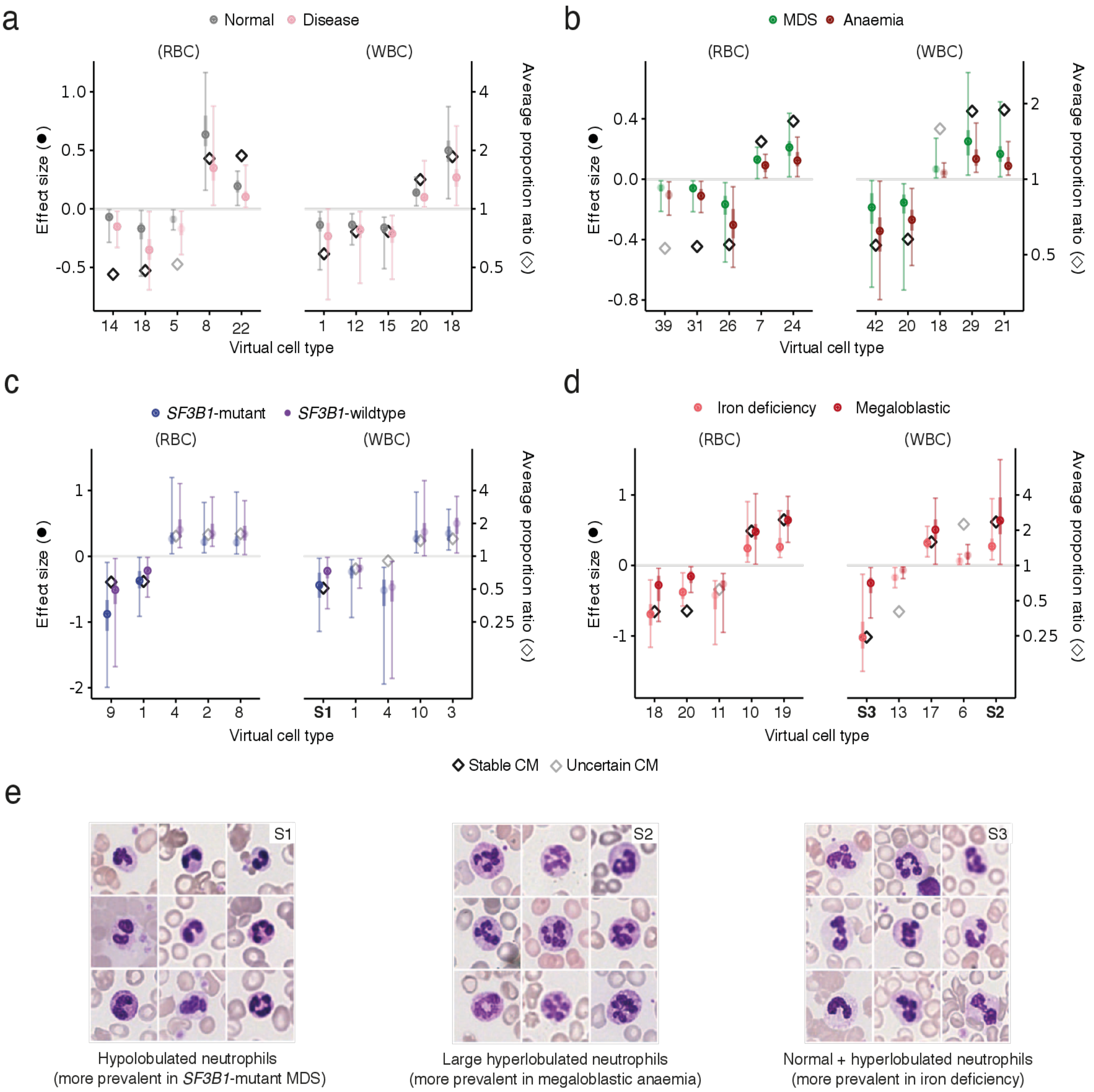

Supplementary Figure S12 - **Effect sizes for the most discriminative computational morphotype (CM) proportions for the single objective Morphotype analysis models and examples. a** — Disease detection (normal vs. disease). Effect sizes above zero are biassed towards the “normal” classification. **b** — Disease classification (MDS vs. anemia). Effect sizes above zero are biassed towards the “MDS” classification. **c** — *SF3B1*-mutant detection (*SF3B1*mutant MDS vs. *SF3B1*-wildtype MDS). Effect sizes above zero are biassed towards the “SF3B1-wildtype MDS” classification. **d** — anemia classification (iron deficiency vs. megaloblastic). Effect sizes above zero are biassed towards the “megaloblastic” classification. For **a-d**, the effect size was calculated as the product of the proportion of each CM and its coefficient in Morphotype analysis. Each point (circle) represents the median effect size and the error bars represent the 90% confidence interval for the effect sizes and corresponds to the left y-axis. Each diamond represents the proportion ratio for each condition and corresponds to the right y-axis (n=9,091,220 RBC and 434,436 WBC across 353 PBS for **a-d**). **e** — Randomly extracted representative examples of CMs validating some conclusions from SO Morphotype analysis.

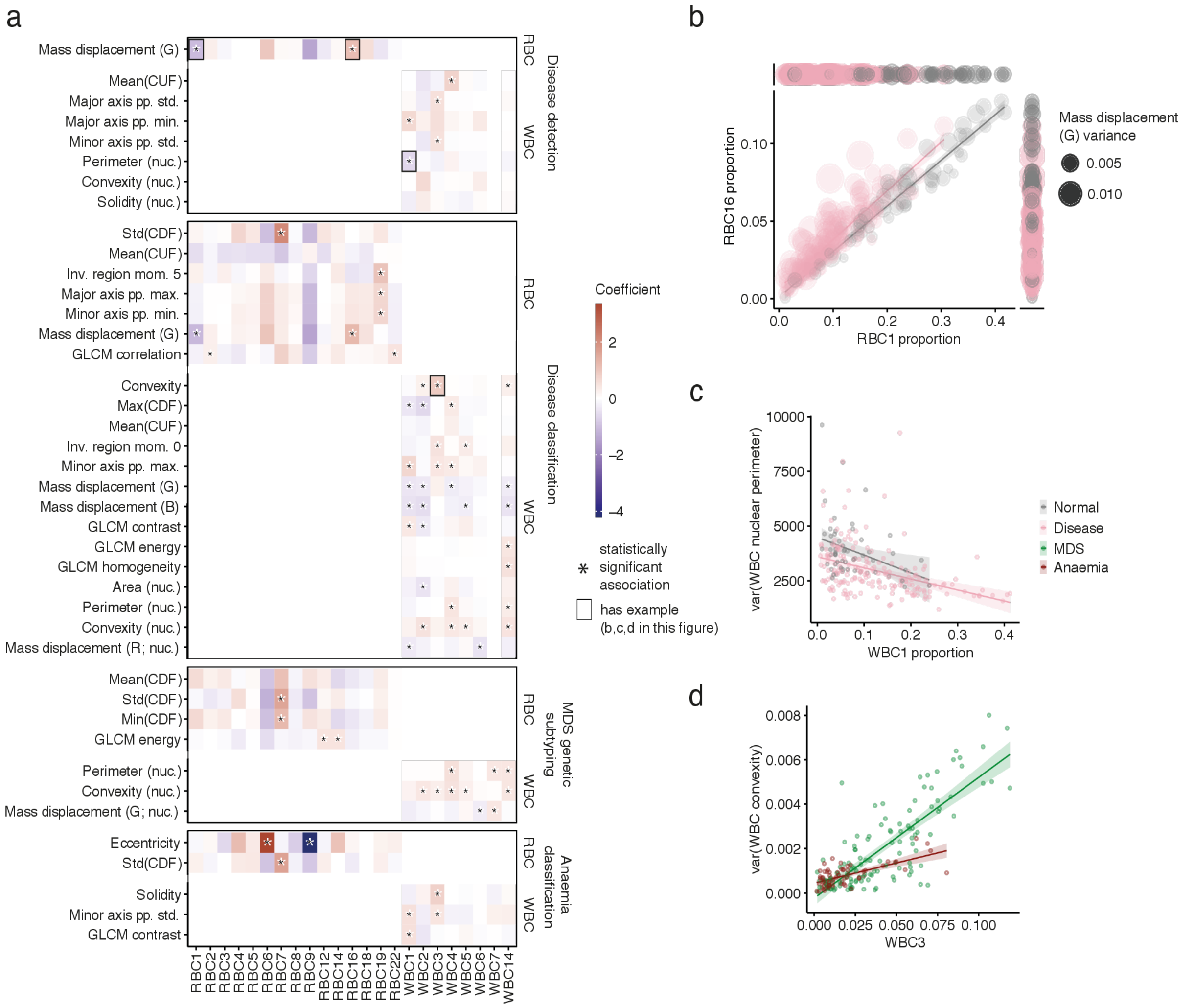

Supplementary Figure S13 - **Computational morphotype proportions capture broad morphometric trends. a** — Partial associations between CM proportions (x-axis) and the variances of different morphometric features (2nd morphometric moment; y-axis). Partial associations (coefficients) between consensual CM and the variance of specific features were calculated according to a linear model that $\mathrm{var}_f = \sum_i^n (\mathrm{cCM}_i \times b_i) + a$, where var_f is the variance of feature f, n is the total number of consensual CM, cCM_i is the proportion of consensual CM i, b_i is the coefficient for proportion cCM_i and *a* is an intercept term. The statistical significance of coefficient estimates was assessed using a two-sided t-test. **b** — Relationship between RCM types 16 and 7 (the latter is A in Figure 5c and Figure 6b) and the mass displacement variance (size of points) stratified by slide classification according to the disease detection model (normal vs. disease). **c** — Relationship between WCM 14 (C in Figure 5b and Figure 6a) and the variance of the WBC nuclear perimeter stratified by slide classification according to the disease detection model (normal vs. disease). **d** — Relationship between WCM 11 (G in Figure 5b and Figure 6a) and the variance of the WBC convexity stratified by slide classification according to the disease classification model (MDS vs. anemia). All reported CMs are stable. CM1-7 in both WCM and RCM are consistent with those reported in previous figures. Other CMs have been arbitrarily numbered.

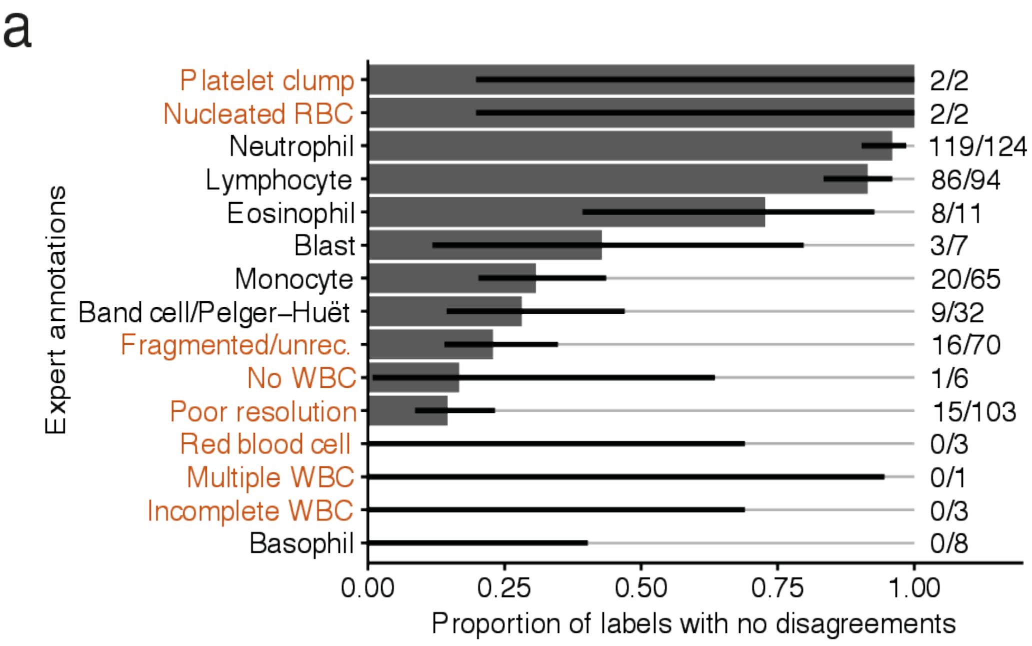

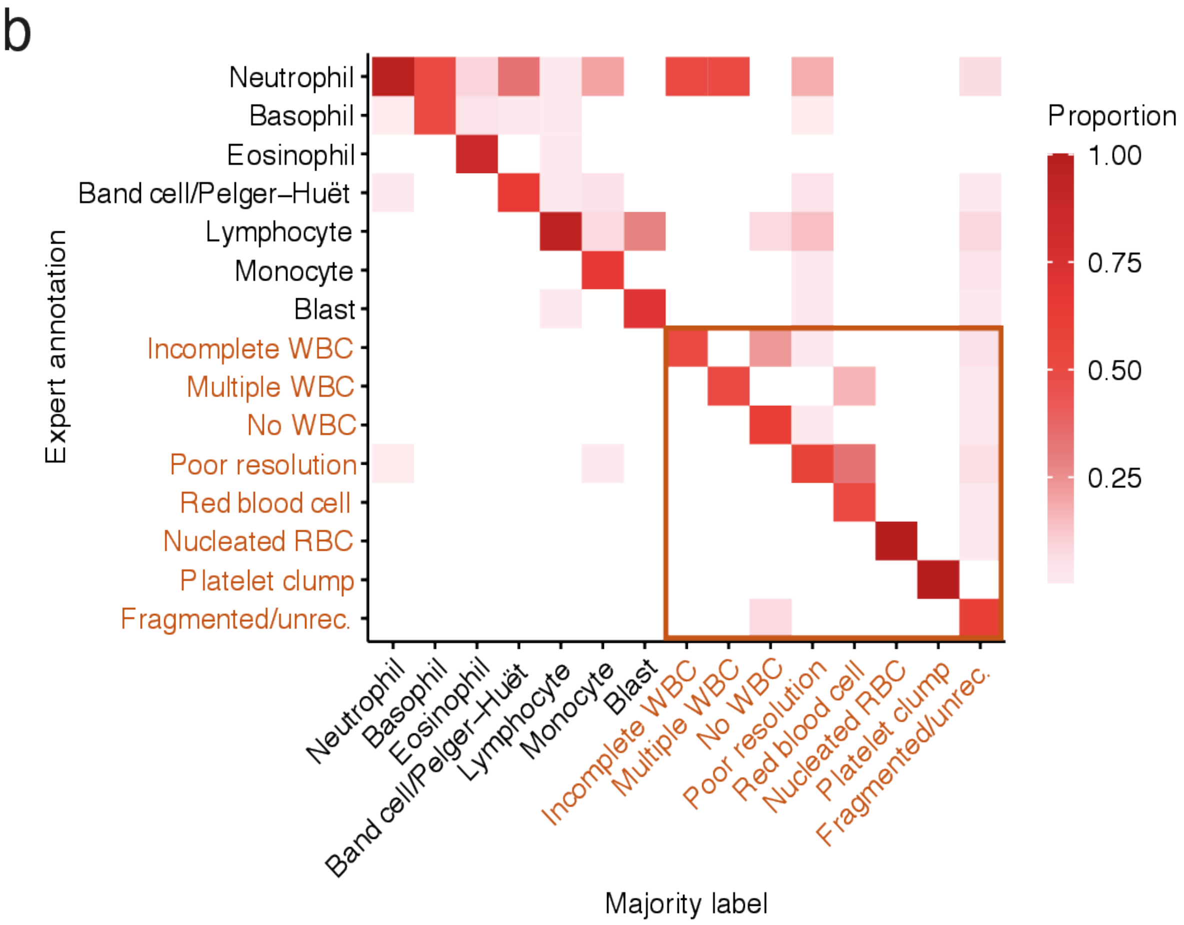

Supplementary Figure 14 - **Concordance between experts in cell annotation. a** — Proportion of expert-labelled WBC with no disagreements when cells were labelled by at least two experts. **b** — Confusion matrix comparing the majority label with the fraction of experts annotating each cell as the majority label.

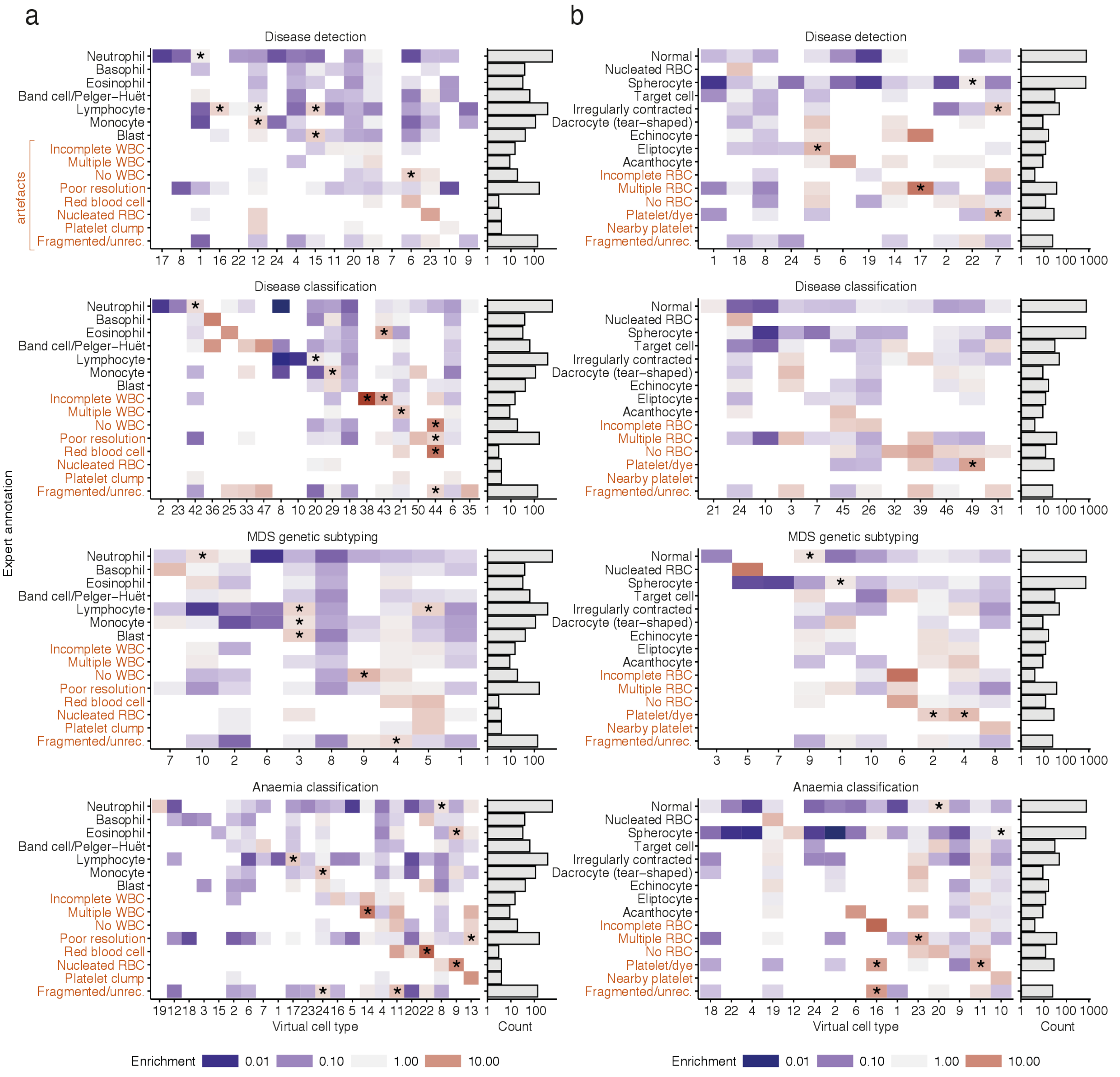

Supplementary Figure 15 - **Computational morphotypes capture expert-annotated cell types with no previous annotation in single objective Morphotype analysis models. a** — Correspondence between WCM types and expert-annotated WBC types (left) and number of annotated WBC types. **b** — Correspondence between RCM types and expert-annotated RBC types (left) and number of annotated RBC types.

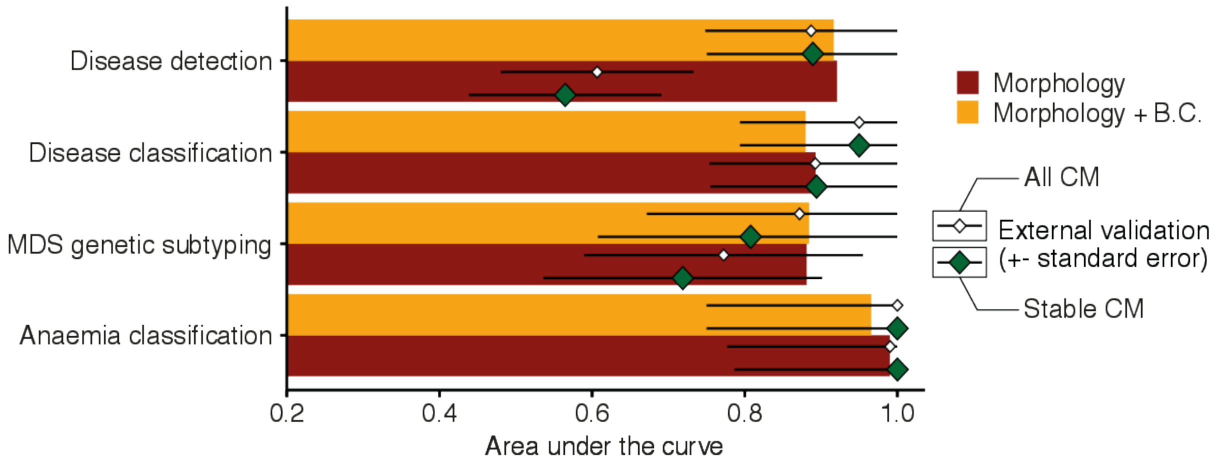

Supplementary Figure 16 - **External validation for single objective Morphotype analysis models.**

Supplementary Methods

Additional details on PBS scanning and visual inspection of PBS quality

Automatically prepared slides from CUH were prepared using a Hematek system. Coverslips were added specifically for the digitalization of the MLL PBS as the routine cytomorphological characterization performed by experts at the MLL is performed using oil immersion microscopy and does typically not require coverslips.

Inadequately prepared (excessive amount of stain) or scanned slides (the whole slide was blurred) were excluded from further analysis. Consequently, 11 slides from the MLL cohort and 1 slide from CUH2 were removed. Finally, for external validation, we excluded 4 PBS in CUH2 since they belonged to the same individual, keeping only one slide per individual.

PBS tiling for prediction

Analysing the entire PBS at once using either RBC or WBC detection protocols is infeasible due to memory constraints — if each pixel is represented as a set of 3 8-bit integers (as is usually the case with images) a PBS from the MLL dataset represents between 11GB and 75GB of required memory; the first layer of the U-Net, with 32 bit output channels, could require 797GB of memory, an unfeasibly high requirement for most computers. For this reason, we consider individual slide tiles which, in our case, are 512×512 pixel for the quality control network and 640×640 pixel ($512 + 64 \times 2$) for RBC and WBC detection. To avoid predicting, at any given point, cells which may be incomplete in a given tile, we exclude all predictions with pixels close to the tile border and to avoid missing these cells use a sliding window with stride 128. Considering that this may lead to detecting the same cell twice as the same object, we exclude a prediction if the center of a cell has already been predicted nearby (within 8 pixels; this prevents Haemorasis from detecting the same cell twice in case it is near the edge of two adjacent sliding tiles). Through this, we estimate the memory requirements for Haemorasis — video or regular RAM — to be 8GB, manageable by most computers and low-to-mid end GPUs.

Machine-learning metrics

To evaluate the performance of the quality control, RBC filtering, WBC segmentation and condition prediction, we first define the possible outcomes of a prediction in terms of their correctness and class:

- True positive (TP) — a prediction is correct and belongs to the positive class
- True negative (TN) — a prediction is correct and belongs to the negative class
- False positive (FP) — a prediction is incorrect and belongs to the positive class
- False negative (FN) — a prediction is incorrect and belongs to the negative class

We can then define the metrics utilized over this work:

- Precision — the ratio of TP to predicted positives (TP + FP): $\frac{TP}{TP + FP}$
- Recall (or sensitivity) — the ratio of TP to total positives (TP + FN): $\frac{TP}{TP + FN}$
- Specificity — the ratio of TN to total negatives (TN + FP): $\frac{TN}{TN + FP}$

- Accuracy — ratio of correct predictions (TP + TN) to all predictions (TP + TN + FP + FN): $\frac{TP+TN}{TP+TN+FP+FN}$
- Intersection over union (IoU) or Jaccard score — the ratio of TP to the union of true positives and predicted positives (TP + FP + FN): $\frac{TP+TN}{TP+TN+FP+FN}$. This can also be stated as the ratio between the intersection of true positives and predicted positives and the union of true positives and predicted positives.
- Average detection metrics — for a given cell, we can consider that a segmentation was successful if the IoU between the annotation and the prediction is above a given threshold. Having several different thresholds allows us to calculate average detection metrics as the area under the curve described by the set of points (metric value, IoU threshold). These metrics include the average precision or recall, and, while the IoU quantifies the performance of the segmentation at the pixel level, these metrics do so at the instance (cell) level.
- F_1 — the harmonic mean of the precision and recall: $\frac{2}{\frac{1}{recall}+\frac{1}{precision}} = \frac{precision \times recall}{precision + recall}$.

  This metric has the advantage of, unlike accuracy, provide a more realistic assessment of predictive performance that is less affected by cases of imbalanced data

We also use the area under the receiver operating characteristic (ROC) curve (AUC). This curve is calculated using the class probabilities p produced by a given predictive model. Using a sequence of thresholds between the minimum and the maximum of p, different values for the sensitivity and specificity are calculated, and a trapezoid curve is constructed where each point corresponds to a threshold value and its coordinates are $\{1 - specificity, sensitivity\}$. The area under this curve (the ROC curve — AUC) is thus a balanced measure of how well a model performs that is insensitive to model calibration.

To calculate the cross-validated AUC we concatenate all predicted probabilities for the validation sets during cross-validation and concatenate them, using the corresponding ground-truth to calculate a final and single cross-validated AUC [1].

Blood cell detection and characterization

Quality control network

This step intends to exclude tiles which are either blurred, have very high cellular density or very low cellular density. While there are relatively simple ways to quantify "blurriness" through a proxy (calculating the variance of the Laplacian, for instance), accurately identifying scenarios of very high or very low cellular density is not as trivial since it requires contextual information (which kinds of cells are visible in the image). Recent work has shown that deep-learning can accurately quantify the blurriness of an image in microscopy settings, outperforming other *ad hoc* metrics devised to quantify blurriness [2].

To remove poor quality tiles, a deep-learning (DL) model was trained on 10,000 randomly selected 512×512 pixel images from 93 slides from the MLL split into training (8,050 tiles from 75 PBS) and testing (1,950 tiles from 18 PBS) sets. Each tile was labeled as “good quality” (or 1, n=2,225) or “poor quality” (or 0, n=7,775). “Poor quality” tiles were tiles where the concentration of cells was too small

(very few/no cells) or too large (high frequency of overlapping cells) or images were blurred (**Supplementary Figure S1**). We fine-tuned the ImageNet-pretrained DenseNet121 [3] (a DL model previously trained on a very large dataset of diverse images, guaranteeing that our model can capture visual features important for human perception) on our training set. We trained our model for 25 epochs with a batch size of 32 and the Adam optimizer with an initial learning rate of 0.00005 that decayed by 90% if the training loss stopped decreasing after an epoch. We trained for 25 epochs as it was observed that the model had converged. During training, each image had a 60% probability of being flipped/rotated, having its brightness, saturation, hue and contrast randomly altered (by 5%, 10%, 10% and 10%, respectively) or of having random JPEG compression artifacts introduced. This makes the training more robust to alteration of PBS preparation and variability in image digitalization. The evaluation of this model was performed on the testing set and using its validation AUC, precision, and recall.

Red blood cell segmentation

The detection of RBC is performed using a routine described in Algorithm 1. For clarity, $CannyEdgeDetector$ represents the Canny edge detection algorithm [4], $getContours$ is a standard routine to detect object contours from a binary image, $drawContours$ is a routine to draw contours on the image and fill them, $getArea$ is a function that calculates the area of a contour or ellipse, $averageColour$ is a function that selects the pixels in an image belonging to a contour and calculates their average value, $fitEllipse$ is a function that fits an ellipse to a contour, $getMajorAndMinorAxis$ is a function that calculates the lengths of the major and minor axes of an ellipse and $isolateObject$ is a function that returns the region of the image containing the RBC and its respective mask. In short, edges and contours were detected using a Canny edge detector [4] and filtered appropriately to ensure that other objects (platelet clumps, groups of RBCs or individual WBCs) are not detected as RBCs. This ensures that only RBC candidates whose morphology is not affected by its neighboring cells are detected. The hyperparameters were selected heuristically with the data used to train the RBC filtering steps; to do this, we2 iteratively varied different thresholds until we could produce a high number of RBC candidates that could be used for a downstream analysis with some additional filtering.

After detection using Algorithm 1, RBC candidates are characterized (details below) and filtered with an extreme tree gradient boosting (XGBoost) model [5], selected due to its fast inference and excellent performance, capable of predicting which candidates are RBC (vs. "not RBC"). To train this model, we picked a random set of 158 2096×2096 pixel tiles from CUH1 and split them into training (109) and testing tiles (48). We then detected, characterized, and annotated 2262 RBC candidates for training ("RBC": 1870; "not RBC": 392) and 1107 ("RBC": 962; "not RBC": 145) for testing. We picked a maximum number of 50 weak estimators and DART boosting [6] for training and validated the model on the testing set. Given the class imbalance (6 of 7 objects belonged to the “RBC” class and classifying all objects as RBC would still constitute acceptable performance), the objective function (importance) of each “RBC” was scaled by a factor of 0.1 during training. Model evaluation was performed using its validation accuracy and F_1. Here, we finally note that this protocol excludes a specific multiple RBC structure characterized as relatively long sequences of clumped RBC (known as “coin stacks” or Rouleaux formations) [7], or cold-agglutinin disease, which are characterized by large amorphous RBC aggregates [8]; however, these formations can arise not only as a consequence of specific clinical conditions, but also as an artifact of slide preparation or inspection of the PBS

outside of the monolayer. Since we wanted our analysis to relate to the clinical experience of practicing hematologists, which focus mostly on the monolayer, we did not attempt to include them in our analysis.

```
Data: image
Result: Set of masked RBC images and their contours S
S ← {};
edgeImage ← CannyEdgeDetector(image);
contours ← getContours(edgeImage);
contourImage ← drawContours(contours);
contourImage ← contourImage − edgeImage;
contours ← getContours(contourImage);
for contour in contours do
  area ← getArea(contour);
  if area > 300 px and area < 2000 px then
    averageColour ← getAverageColour(image; contour);
    if averageColour > 170 and averageColour < 220 then
      ellipse ← fitEllipse(contour);
      majorAxis, minorAxis ← getMajorAndMinorAxis(ellipse);
      areaEllipse ← getArea(ellipse);
      if areaEllipse < 1500 px and areaEllipse > 300 px and majorAxis/minorAxis < 1.5 then
        isolatedCellImage; isolatedCellMask ← isolateObject(image; contour);
        append {isolatedCellImage, isolatedCellMask, contour} to S;
      end
    end
  end
end
```

Algorithm 1: Red blood cell segmentation and detection algorithm.

U-Net models training and nuclei segmentation

Our WBC segmentation and detection algorithm is based on U-Net, a DL model for biological image segmentation [9]. As this model requires annotations at the pixel level, we labeled WBCs in 158 2096×2096 pixel images from CUH1, comprising a total of 2,853 annotated WBC including 2,747 completely present WBC. Two additional images from MLL and 30 images from CUH2 (containing 60 and 69 WBC, respectively) were annotated to test the generalization of our model. Different architectures were trained on 512×512 pixel tiles obtained from the original 2096×2096 pixel images with a learning rate of 0.001 and a weight decay of 0.005 using a weighted cross-entropy loss and the Adam optimizer [10], whilst making extensive use of data augmentation (**Supplementary Table S3**) and used the Jaccard index (IoU) to assess the performance of these models. The reason behind our extensive data augmentation protocol is backed by recent findings that show how using extensive (and even unrealistic) image augmentation techniques leads to improved performance in histopathology [11]. To assess the performance at the instance (cell) level, we also calculate the

average precision and recall at 10 different IoU thresholds, defined linearly between 0 and 1. We tested the impact of test-time augmentation (TTA) [12] and prediction post-processing on performance. To assess whether network depth (the number of channels in each convolutional layer) had an impact on output; for this, different instances of a U-Net model were trained where the depth of each layer was multiplied by 1.0, 0.5 and 0.25 and rounded to the nearest integer (this varies the number of features that a model seeks to identify and use to explain the data). During training, we make extensive use of data augmentation in order to make our models more robust to other images/datasets.

Finally, we segmented the WBC nuclei by clustering WBC pixels using k-means clustering (k=2) as described in other works [13]. Summarily, we cluster pixels belonging to WBC into one of two groups and, as nuclei stain darker than the rest of each WBC, this enables easy assignment of each pixel to the darkest (nucleus) or brightest (cytoplasm) parts of the cell.

Data augmentation and prediction-time enhancements to U-Net predictions

To train the U-Net models we used data augmentation to achieve the best possible results — using the original 2096×2096 pixel images, 512×512 pixel tiles are randomly extracted after rotating the image at a random angle between 0 and 89 degrees. Next, each image is distorted using an elastic transform as implemented in albumentations [14], following random image flips and/or rotations and perturbations to the brightness, saturation, hue and contrast of the image. Finally, we randomly add salt and pepper and Gaussian noise to each image, which can also be slightly blurred (using a standard Gaussian blur). The probabilities and parameters for these augmentations are presented in **Supplementary Table S3**.

To ensure the best possible performance using our U-Net model, we used test-time augmentation (TTA) and prediction post-processing. The former is characterized as rotating and flipping the input image in order to derive a consensus prediction from the several outputs, whereas the latter was a custom process. Particularly, for TTA, we rotate the image by 90, 180 and 270 degrees (4 inputs) and then rotate the outputs to the original image orientation, calculating the average pixel probability across the 4 outputs [12]. Prediction post-processing is done by removing objects detected as WBC whose size lies outside the expected distribution for WBC sizes, and filling small convex hull defects. The expected size distribution for WBC was determined as the lower and upper bound of the WBC used for segmentation rounded up or down (respectively). This corresponds to 1000 and 8000 pixels when the resolution is 0.2268 micrometers/pixel or 51.4 squared micrometers and 411.5 squared micrometers, respectively.

Morphometric characterization

After detecting and segmenting each cell, we calculate a set of cytomorphological descriptors for each one. The descriptors used here and described in **Supplementary Table S5** were implemented in a custom Python script, but the large majority are also present in widely used bio-image analysis software programs [15,16] or described in publications reviewing morphometry in image analysis [17]. We characterize every RBC and WBC, as well as the nucleus of each WBC (**Supplementary Table S5**). Overall, we capture 53 features for each WBC (42 for the cellular characterization and 11 for the nuclear characterization) and 42 features for each RBC.

To account for different resolutions (0.2268 micrometers/pixel for Hamamatsu NanoZoomer 2.0; 0.2517 micrometers/pixel for Aperio AT2) we rescale the images in CUH2 by a factor of 1.1098 prior to cellular characterization.

We calculate morphometric moments, the parametric characterizations of the distribution of each feature within each PBS, as the mean and variance of each RBC and WBC feature on each PBS. Using this, we describe the cytomorphological landscape of each PBS using 190 features ($42 + 53 \times 2$).

Online visualizer of morphotypes

To assist in the visualization of different morphotypes, a two-dimensional UMAP [18] was calculated for both 10,000 RBC and 10,000 WBC using their morphometric features. Having these features, we also assigned each the morphotype of each cell and coloured each cell (each point in the UMAP) according to their morphotype. The platform was implemented in native HTML and Javascript with recourse to the D3 visualization library [19] and is available in https://josegcpa.github.io/haemorasis-umap. By using D3 we ensure that the visualization are relatively fast despite making the visualization of 20,000 cells possible.

Blood counts and morphometric moment preprocessing and feature importances in glmnet

Before training the elastic net models, we standardize variables and impute missing values in the blood counts using the median value of each fold (a total of 139 blood counts were inferred for 49 individuals). To assess feature importance in glmnet, we use the absolute value for the standardized coefficients. To calculate the feature importance for the 5 relevant groups of features — for the means and variances of WBC (2) and RBC (2) and the blood counts (1) — we first calculate the effect of each group of variables by summing the effects of the standardized variables composing it for each individual. Then, we calculate the total explained variance by each group of features by calculating the sum of the rows in the covariance matrix between all 5 groups of features and dividing it by the total explained variance (sum of all elements of the covariance matrix).

Morphotype analysis — model specification and optimization

Motivation. Typically, when diagnosing hematological conditions from a PBS, an expert focuses on identifying abnormal cell types and quantifying their relative prevalence [20]. In more abstract terms, the expert classifies objects in a set to quantify the relative prevalence of abnormalities and classifies the set based on this. This is essentially a problem of multiple instance learning (MIL), a machine-learning field that focuses on classifying a set of objects based on its composition. Some MIL methods combine this group classification task with the auxiliary task of learning how to map objects to a vocabulary (set of terms) and uses the frequency of instances in each term to predict the classification of a group [21].

Model specification. A given PBS is characterized as a set of n cells $O \in R^{n \times f}$, where f is the number of morphological features characterizing each cell. We want to assign each of the n cells to one of v CM, such that $V = softmax(O \times \theta_V, axis=1)$, where $V \in R^{n \times f}$ is the membership (between 0 and 1) of all cells to each CM and $\theta \in R^{f \times v}$ are the parameters of the softmax regression assigning each cell to one of v CM. The computational morphotype (CM) composition of the slide $C \in \Delta^v$,

where Δ^v is the simplex of order v, is then calculated as $C = mean(V, axis=0)$. C is then used to calculate the probability that a PBS belongs to an individual with a given disease d as $P(d \vee C) = sigmoid(\theta_C \times C)$, where θ_C parametrizes the weights of each CM for the classification probability $P(d \vee C)$.

We characterize the RBC $R \in R^{r \times f}$ and WBC $W \in R^{w \times f}$ in a slide S as separate entities, where r and w are the number of RBC and WBC, respectively, and f and g are the number of features characterizing each RBC and WBC, respectively. Then, the RBC and WBC CM (RCM and WCM, respectively) compositions of each slide are calculated separately (with $\theta_{R,RBC} \in , R^{f \times v}$ and $\theta_{W,WBC}, \in R^{g \times v}$, respectively) and use them to calculate $P(d \vee C_{RBC}, C_{WBC}) = sigmoid(\theta_{C,RBC} \times C_{RBC} + \theta_{C,WBC} \times C_{WBC})$. This formulation also enables the use of additional information, particularly blood counts ($counts$, used earlier in this chapter) such that $P(d \vee C_{RBC}, C_{WBC}, counts) = sigmoid(\theta_{C,RBC} \times C_{RBC} + \theta_{C,WBC} \times C_{WBC} + \theta_{counts} \times counts)$.

Model optimization. We are interested in the concurrent optimization of $\theta_{V,RBC}$, $\theta_{V,WBC}$, $\theta_{C,RBC}$, $\theta_{C,WBC}$ and θ_{counts} such that Morphotype analysis models classify cells into condition-specific CM while learning how to predict conditions from specific PBS. For this reason, we used stochastic gradient descent to optimize Morphotype analysis models, assuming that there are an equal number of WCM and RCM. We use a cross-entropy loss and L_2 regularization where $\lambda = 0.2$.

We also train multiple objective models which optimize all four tasks using the same set of CM. For this, in each training step and for each of the four tasks, a set of relevant PBS is sampled and the cross-entropy is calculated. All four loss values are summed after multiplying them by a vector sampled from a Dirichlet distribution with concentration [1,1,1,1] as proposed in [22] to approximate Pareto optimality (i.e. no improvement can be done to one task without making a different task worse [23]) and backpropagate this value.

To select the best hyperparameter values (number of CM) in each setting we used the cross-validated AUROC. To get these cross-validated scores, each model is trained over 5 folds and the average value and standard error of each metric is calculated. In each fold, a model is optimized for a maximum 15,000 steps with a learning rate of 0.01 and a weight decay of 0.25. We used weighted cross-entropy with the Adam optimizer [10]. The weight for the positive class is calculated as $1 - \frac{no.\,of\ elements\,in\,the\ positive\,class}{no.\,of\ total\,elements}$. Additionally, the learning rate is decreased after the training loss stagnates for at least 100 steps by a factor of 0.5. If the learning rate reaches a value of 0.000001 (smaller than the initial learning rate by 4 orders of magnitude) training is stopped. Each training iteration samples 500 WBC and 500 RBC. This may lead to a situation where bags of cells with a smaller amount of information (fewer cells) contribute equally to those with a large amount of cells. To minimize this, each instance is weighted with w_{avail}, calculated from the number of available RBC m and WBC n such that $w_{avail} = 1 + \frac{minimum(500, m) + minimum(500, n)}{1000}$. w_{avail} will be closer to 1 when fewer cells are available and, if more than 500 WBC and 500 RBC are available, it will be 2.

We trained models for each task using different numbers of CM — $[25,50]$ for MO Morphotype analysis and $[10,25,50]$ SO Morphotype analysis — assuming that both WBC and RBC can be clustered into the same number of CMs, and whether blood counts (WBC counts (cells/μm), haemoglobin concentration (g/dL) and platelet counts (platelets/μm)) improved the classification performance of Morphotype analysis. All variables were standardized (mean = 0 and standard deviation = 1).

Predictions using stable CMs. Since we use a single solution (i.e. a single fold) for inference and testing this can lead to a generic problem of determining a latent space (CMs, in our case) — oftentimes, the mapping between input and latent space becomes too specific for the training data on each fold and underperforms on other datasets by detecting CM which correspond to artefacts rather than to clinically-relevant cytomorphologies. For this reason, we use only CM which are stable across the 5 cross-validation folds. To define these CM, we sample 500 WBC and RBC from each PBS with replacement (172,000 WBC and 172,000 RBC in total) and calculate their CM probability (the probability that each cell belongs to a specific CM) for the best performing and other folds ($C_{\frac{WBC}{RBC},best}$and $C_{\frac{WBC}{RBC},k}$, respectively, where $best$ corresponds to the best-performing fold and $k \in \{1,2,3,4\}$ corresponds to other folds). We then calculate the correlation R between all CM in the best-performing fold and all CM in other models. This measures how similar a CM in the best performing fold is to those in other folds. Next, we match CM in the best-performing fold to those in other folds by considering, for each CM in each combination of best and k, the CM pair with the highest R. Finally, we consider that a cell in best is stable whenever there are three or more matches where $R > R_{thresh}$ (in our case, $R_{thresh} = 0.5$). In other words, a CM is stable whenever it is sufficiently correlated with any other CM across most folds trained with different subsets of the same data.

Inference using stable CMs is then performed by setting the proportions of all non-consensual CM to 0 and distributing the proportions across the remaining, consensual CM. While the ideal case scenario would be to retrain the model with the subset of consensual CM, we only assess performance using the AUC, which does not depend on the absolute value of the classification probability, depending rather on the ranking of different classification probabilities.

Stable CM labelling in this manuscript. In **Figure 4**, **Figure 5**, **Supplementary Figure S10** and **Supplementary Figure S11** we show only stable CMs and enumerate them accordingly (WBC CM labels and RBC CM labels correspond with each other across these figures). In **Figure 6**, where we show all CMs enriched in an expertlabelled cell type, we label stable CMs as **CMx**, where **x** corresponds to the numbers in the figures previously stated in this paragraph, while other other CMs are labelled using letters to avoid confusion. In **Supplementary Figure S11**, non-stable (uncertain) CMs are labelled as **Ux**.

Associations between CM proportions and variance. To calculate how the relative prevalence of different CM is associated with changes in the variance of specific morphometric features, we fit, for important variance features in the glmnet models (non-zero variance features), the linear model $var_f = \sum_i^n (cCM_i \times b_i) + a$, where var_f is the variance of feature f, n is the total number of consensual CM, cCM_i is the proportion of consensual CM i, b_i is the coefficient for proportion cCM_i and a is an intercept term. To make each term more comparable, we normalize variance and

CM proportions independently as described in "Morphometric moment preprocessing and feature importances in glmnet" in the **Supplementary Methods**. To assess whether these coefficients (associations) are statistically significant, we use t-tests and correct for multiple testing using the Bonferroni method considering a threshold of $\alpha = 0.05$ ($\alpha_{corrected} = \frac{0.05}{405\, tests} = 0.000123$). To calculate the feature densities shown in **Figure 5**, we use a subset of 31,119 RBC and 31,884 WBC from the MLL cohort.

Expert annotation of blood cells

RBC were observed individually (in a 256×256 image centered on a single RBC of interest, noted with a green bounding box, were shown to each hematologist) and classified into one of the following classes: "normal", "nucleated RBC", "spherocyte", "target cell", "irregularly contracted RBC", "dacrocyte", "keratocyte", "echinocyte", "eliptocyte" and "acanthocyte", or into: "incomplete RBC", "multiple RBC", "no RBC", "platelet/dye", "nearby platelet" and "unable to tell". WBC were observed individually (in a 128×128 image containing, in practical terms, only the WBC) and annotated with one of the following classes: "neutrophil", "basophil", "eosinophil", "band cell", "lymphocyte", "monocyte", "blast", with the artefacts "incomplete WBC", "multiple WBC", "no WBC", "poor resolution", "RBC", "nucleated RBC", "reticulocyte", "fragmented/unrecognizable" and "platelet clump".

Setting up Haemorasis

As a Docker container. This is the recommended route as it requires only a Docker installation [24] (also easily transferrable to Singularity) and prevents possible conflicts. The commands to be followed are standard for prebuilt Docker images, i.e. a pre-built image should be pulled from Docker Hub — in this case, `docker pull josegcpa/blood-cell-detection:latest`. This image contains the necessary dependencies and scripts to run Haemorasis.

From the git repository. This is not the recommended route as several dependencies have to be installed and errors/incompatibilities are more likely. This route requires the user to have Git, as well as Python 3.6.8 installed. Setting up Haemorasis should be done as follows, assuming the user is operating a UNIX based system and from the command line:

1. Clone the https://github.com/josegcpa/haemorasis repository to the machine where Haemorasis is to be run (`git clone https://github.com/josegcpa/haemorasis`)
2. Install the required packages using pip (`pip install -r requirements-pipeline.txt`)

Running Haemorasis

Simplified explanation of Haemorasis. Haemorasis is composed of a set of 7 steps, orchestrated using Snakemake:

1. The model checkpoints and parameters are downloaded if they are not available
2. Quality control of the PBS — each 512×512 pixel tile is quality controlled to filter out tiles with excessive/deficient cellular density and/or poor resolution (output stored as `{output.directory}/_quality_control/{slide. id}.h5`)
3. Segmentation of WBC and RBC — segmentation coordinates are stored in hdf5 format, with a dataset for each cell (output stored as

{output.directory}/_segmented_wbc/{slide.id}.h5 and {output.directory}/_segmented_rbc/{slide.id}.h5, respectively)

4. Morphometric characterization of WBC — morphometric features are stored in hdf5 format, with a dataset for each cell (output stored as {output.directory}/_aggregates_wbc/{slide.id}.h5)
5. Morphometric characterization of RBC — morphometric features are stored in hdf5 format, with a dataset for each cell (output stored as {output.directory}/_aggregates_rbc/{slide.id}.h5)
6. Annotation of RBC in geojson format — these annotations can be loaded into QuPath (output stored as {output.directory}/_annotations_rbc/{slide.id}.h5)
7. Annotation of WBC in geojson format — these annotations can be loaded into QuPath (output stored as {output.directory}/_annotations_wbc/{slide.id}.h5)

Steps 4 and 5 are run in parallel, as well as steps 6 and 7. In the examples above, {slide.id} is the basename of the slide until the first dot (if the slide path is /homes/user/slide_32.0.1.tiff then {slide.id} is slide_32). The output directory ({output.directory}) is the directory which will contain the output from the analysis.

Pipeline usage — single slide. To run Haemorasis for a single slide, the run-slide.sh script is used, where two key options are necessary:

- -i — the path to a slide
- -o — the path where the output will be stored

As such, an example of running Haemorasis is, for a slide in /homes/user/slides/slide_a.tiff and the output directory in /homes/user/output is sh run-slide.sh -i /homes/user/slides/slide_a.tiff -o /homes/user/output. sh run-slide.sh -h displays other available options.

Pipeline usage — folder of slides. To run Haemorasis for a folder of slides, the run-folder.sh script is used, where three key options are necessary:

- -i — the path to a folder containing slides
- -f — the file extension for the slides (.tiff, .svs, .ndpi and other formats supported by Openslide [25])
- -o — the path where the output will be stored

As such, an example of running Haemorasis is, for a folder of slides in .tiff format in /homes/user/slides and the output directory in /homes/user/output is sh run-folder.sh -i /homes/user/slides -f tiff -o /homes/user/output. sh run-folder.sh -h displays other available options.

Adaptation to Docker container. Please note that the examples provided below do not incorporate the Docker command line interface syntax. Additionally, for our use case, where both input and output should be or become locally available, bind mounts are required for the input and output.

As such, to run the Haemorasis command sh run-slide.sh -i /homes/user/slides/slide_a.tiff -o /homes/user/output inside our pulled Docker image josegcpa/blood-cell-detection:latest, one should run docker run -v /homes/user/slides:/slides -v /homes/user/output:/output josegcpa/blood-cell-detection:latest sh run-slide.sh -i /slides/slide_a.tiff -o /output. For more information on Docker

please consult the Docker documentation in https://docs.docker.com/ and to get started with Docker please refer to the Get started tutorial in https://docs.docker.com/get-started/.

Visualizing detected cells. QuPath is a popular software for digital pathology images [26]. Haemorasis has the advantage of producing GeoJSON files with RBC and WBC annotations which can be easily visualized with slides using QuPath in `{output.directory}/_annotations_rbc` and `{output.directory}/_annotations_wbc`, respectively.

Details regarding software packages and their versions

During this work we have made extensive use of different Python (version 3.6.8) [27] and R (version 3.6.1) [28] packages. We provide details regarding their application below, with versions noted with "==" after the package name. For instance, to refer to version 1.9 of package X we write "X==1.9".

Python packages

Numerical manipulation and scientific calculations were performed using numpy [29] and scipy [30]. For image manipulation at the pixel level, we used Pillow==7.2.0 [31], scikit-image==0.17.2 [32] and opencv-python==4.4.0.44 [33], as well as albumentations [14] for elastic network deformation (a type of data augmentation). openslide-python==1.1.2 [25] was used for manipulation and read-write operations for digitized PBS files. For machine-learning, we made use of scikit-learn==0.23.2 [34] and xgboost==1.3 [5] (through its scikit-learn interface), as well as Tensorflow==2.0 [35] and PyTorch==1.10.1 [36]. Intermediate data storage was handled using the hdf5 format through h5py==2.10.0 [37]. Dataframe manipulation was handled using pandas==1.1.5 [38]. Finally, we made use of snakemake [39] for workflow management.

R packages

Dataframe manipulation in R, as well as comma-separated values files read-write operations were handled using tidyverse==1.3.1 [40] and base R. Machine-learning analyses with elastic network models were done using glmnet==2.0-16 [41] and caret [42], with metric analysis performed using MLmetrics==1.1.1 [43] and pROC==1.18.0 [44]. Plotting was largely done using ggplot2==3.3.5 [45] with ggpubr==0.4.0 [46] and ggsci==2.9 [47], as well as cowplot==1.1.1 for plotting panels. General statistical tests and robust R testing was performed using MASS==7.3-51.3 [48] Dunn testing was done using the dunn.test==1.3.5 package [49]. Finally, Uniform Manifold Approximation and Projection (umap) was performed using umap==0.2.7.0 [50].

Supplementary Results

Performance of the quality control network

To train this model, we manually annotated a dataset from the 8,050 tiles from the MLL PBSs into "good" or "poor" quality tiles, and validated our model on a dataset of 1,950 tiles from distinct MLL cohort PBSs. The model performed well and comfortably within what we required for this filtering step (validation $AUC = 93.4\%$, $recall = 82.2\%$ and $precision = 85.2\%$). Of note, most errors were not clear mistakes, but represented tiles of borderline quality, with the predicted "good" quality regions corresponding to the monolayer (**Supplementary Figure S1a,b**).

U-Net performance

U-Net performance is summarized in **Figure 2b** and **Supplementary Figure S2**. For the best performing model – U-Net with depth=0.5 and TTA and prediction post-processing – the average intersection between ground truth and prediction over the union of both (IoU) of 84.4% for CUH1 and 88.9% for both CUH2 and the MLL cohort. Additionally, we observe that using the post-prediction processing (**Supplementary Methods**) greatly reduces the amount of false positives (defined as predicted objects with no overlap with any ground truths) with a relatively small reduction in the recall (average reduction in recall = 3.9% and average improvement in precision = 31.7%). The fact that a smaller version of the U-Net (fewer parameters) was the best performing model for WBC segmentation is a likely consequence of a decrease in the chance of overfitting (**Supplementary Figure S2**). Interestingly, we observe that the U-Net with depth multiplier=0.25 severely underperforms in CUH2; we postulate that this is because a smaller amount of extracted features leads to a lack of generalization to different scanners (slides in CUH2 was scanned with different scanners from those used for slides in CUH1 and MLL).

RBC filtering performance

RBC filtering ensured that non-RBC objects in PBSs were removed. Our model, based on XGBoost [5] and morphometric characterization of RBC candidates, was validated on an independent test set and showed high discriminative ability with validation $accuracy = 90.6\%$ and $F_1 = 94.2\%$ (F_1 is a good metric for imbalanced data).

Estimating the analysis time per RBC and WBC for Haemorasis

To better understand the applicability of Haemorasis, we estimate how much time would be necessary for the analysis of each WBC and RBC. We observe that run time is highly variable and highly dependent on the number of detected cells, as described in **Supplementary Table S6** - while slides in the first run time decile took, on average, 2,445 seconds (approximately 40 minutes) to run, those in the upper decile took an average time of 81,335 seconds (approximately 22 hours and 30 minutes). However, it should be highlighted that this is highly dependent on the average time number of cells (WBC and RBC) and number of “good” quality tiles. Even more importantly is the fact that, for any given quantile, we detect a relatively high number of WBC and RBC. This led us to assess, rather than the average run time, the average time per detected and characterised cell, calculating a regression where the number of “good” quality tiles, the total number of tiles, the number of WBC and the number of RBC are independent variables and Haemorasis run time is the dependent variable. We do not model an intercept term as, in theory, if the total number of tiles is 0, all other variables (including the run time) should also be 0.

While the coefficients for the total number of tiles and "good" quality tiles were statistically non-significant, this shows that, on average, each WBC takes 6.38 seconds ($p=6*10^{-6}$ for a two-sided t-test) and each RBC takes 0.55 seconds ($p=4*10^{-4}$ for a two-sided t-test), implying that analyzing substantially smaller amounts of WBC and RBC (200 each) would take, on average, 23 minutes. Here, we use 200 cells as it is comparable to the number of analyzed WBC in PBS when diagnosing MDS per standard diagnostic criteria [51]. The remaining question, however, is whether or not this would negatively impact the performance of our models, i.e. whether prediction using fewer cells could lead to reduced performance. We test this using a subset of 200 WBC/RBC and a subset of 500 WBC/RBC. As shown in **Supplementary Table S7**, there is only a considerable decrease in the AUC of the morphology-only models for the disease detection models; however, including the blood counts leads to no observable decrease in performance. Hence, we conclude that, while this work focused on the analysis of very large collections of cells, this could be applicable even if only a small fraction of cells (i.e. subsets of 200 or 500 WBC/RBC) were analysed.

Analysis of the relevance of individual morphometric features in prediction

To assess whether individual morphometric features (Methods) could be used to discriminate between conditions, we first calculated, for each slide, its morphometric moments — the mean and variance of each morphometric feature obtained for all WBC and RBC — and then assessed whether relevant differences were present between different conditions using a Kruskal-Wallis test. Upon statistical significance after controlling for false-discovery (95%), we determine their utility on specific comparisons using Dunn-Bonferroni tests. We observed that, for WBC, the morphometric moments of 60.0% (36/60 for *SF3B1*mut MDS detection) to 81.7% (49/60 for disease detection) of features are useful to discriminate between different conditions, while for RBC these proportions were between 48.8% (20/41 for disease classification) and 90.2% (37/41 for anemia classification; **Supplementary Figure S6e,h**).

Avoiding demographic and blood count confounders in determining morphometric trends

As illustrated by Bruck *et al.*, bone marrow cytomorphology is associated with age, sex and some blood counts [52]. In our case, this can be critical – for instance, it is well known that platelets are higher in *SF3B1*-mutant MDS when compared with other MDS subtypes [53]. If we only used morphological features to predict *SF3B1* and used the feature importances or morphotypes stemming from this analysis to determine how cytomorphology is altered in *SF3B1*-mutant MDS, we could easily mistake clinically-relevant changes to cytomorphology with some other, non-MDS upstream cause responsible for both increased platelet counts and changes in cytomorphology. However, it should also be noted that excessively controlling for these possible confounders can also be problematic and lead to excessively conservative models – it is also possible that one observes an association between cytomorphology and blood counts or other factors because they are both being caused by the same, clinically-relevant condition. In this case, if we remove features associated with the blood count we may be diluting the real and clinically-relevant cytomorphological signal from our data.

Taking the previous paragraph into consideration and to reduce the effect of confounding variables on our conclusions regarding clinically relevant cytomorphological trends/morphotypes, we focus the large part of our morphometric moment and morphotype analyses on models which control for

different blood count levels. More concretely, we include blood counts in the linear models considering morphometric moments as an additional variable (**Methods; Supplementary Methods**) and we also include blood counts in the Morphotype analysis by including the blood count values in the "classify conditions" step in **Figure 3** (and further explained in the **Methods** and **Supplementary Methods**). This prevents either model from learning redundant information (i.e. information that is also contained in blood counts). This is particularly relevant as, upon training elastic-net regularized linear regression models with a Gaussian response to predict WBCC, Hb and Plt using 5-fold cross-validation, we observe statistically significant (albeit small) R^2 estimates between predicted and observed values – R^2=0.027 (p=0.02) for WBCC, R^2=0.086 (p=$3*10^{-5}$) for Hb and R^2=0.16 (p=10^{-8}) – further motivating the inclusion of blood counts in our cytomorphology models. To reduce the effect of clinical status on this assessment, we used only WBS belonging to individuals diagnosed with MDS.

Finally, and particularly considering that we do not include age or sex in our models, we performed similar analyses as those described in the previous paragraph for age and sex (for age we trained an identical elastic-net regularized linear model with a Gaussian response, whereas for sex we used a binomial response). We find no statistically significant association between the predicted and observed age (R^2=0.005; p=0.3), and observe that the AUC for sex prediction is not statistically significant (AUC=56.2%, DeLong $CI_{95\%}$=[48%,64.4%]). Both of these factors indicate that morphometric features are not associated with age or sex.

Supplementary tables

Table S1: Blood counts, age, sex and clinical classification of individuals in the MLL cohort. NA = not available.

Slide ID	WBC/uL	Haemoglobin (g/dL)	Platelet/uL	Clinical classification	Clinical classification (simplified)	Sex	Age
I-1	8400	12.9	219000	Normal finding	Normal	male	[40-45[
I-10	NA	NA	NA	Normal finding	Normal	female	[85-90[
I-11	7390	18.3	246000	Normal finding	Normal	male	[30-35[
I-12	NA	NA	NA	Normal finding	Normal	female	[55-60[
I-13	NA	NA	NA	Normal finding	Normal	male	[20-25[
I-14	NA	NA	NA	Normal finding	Normal	female	[75-80[
I-15	7200	16.6	496000	Normal finding	Normal	male	[60-65[
I-16	6900	15.2	325000	Normal finding	Normal	female	[60-65[
I-17	8000	12.1	176000	Normal finding	Normal	female	[30-35[
I-18	9700	13.9	366000	Normal finding	Normal	male	[40-45[
I-19	9960	14.3	375000	Normal finding	Normal	female	[60-65[
I-2	5200	13.6	218000	Normal finding	Normal	female	[50-55[
I-20	4100	14.5	191000	Normal finding	Normal	female	[65-70[
I-3	5400	14	217000	Normal finding	Normal	female	[70-75[
I-4	4500	14.4	195000	Normal finding	Normal	male	[70-75[
I-5	6400	13.1	235000	Normal finding	Normal	female	[30-35[
I-6	8100	12.9	181000	Normal finding	Normal	male	[55-60[
I-7	7500	16.9	270000	Normal finding	Normal	male	[45-50[
I-8	10200	14.2	206000	Normal finding	Normal	female	[15-20[
I-9	NA	NA	NA	Normal finding	Normal	male	[30-35[
II-1	6000	11.3	148000	MDS SF3B1 mutated	MDS	male	[80-85[
II-10	6600	10.7	240000	MDS SF3B1 mutated	MDS	female	[70-75[
II-11	4300	13.4	210000	MDS SF3B1 mutated	MDS	female	[80-85[
II-12	3080	11.3	29000	MDS SF3B1 mutated	MDS	male	[70-75[
II-13	5200	11.3	260000	MDS SF3B1 mutated	MDS	male	[55-60[
II-14	4400	10.8	110000	MDS SF3B1 mutated	MDS	female	[80-85[
II-15	5450	10.7	235000	MDS SF3B1 mutated	MDS	female	[70-75[
II-16	NA	NA	NA	MDS SF3B1 mutated	MDS	male	[70-75[
II-17	NA	NA	NA	MDS SF3B1 mutated	MDS	female	[70-75[
II-18	3900	8.7	336000	MDS SF3B1 mutated	MDS	female	[55-60[
II-19	13300	9.1	679000	MDS SF3B1 mutated	MDS	male	[85-90[
II-2	13300	11.1	354000	MDS SF3B1 mutated	MDS	male	[75-80[
II-20	6600	10.6	437000	MDS SF3B1 mutated	MDS	female	[60-65[
II-21	4400	10	160000	MDS SF3B1 mutated	MDS	male	[75-80[
II-22	8200	11.2	350000	MDS SF3B1 mutated	MDS	male	[55-60[
II-23	7500	8.7	80000	MDS SF3B1 mutated	MDS	male	[80-85[
II-24	NA	NA	NA	MDS SF3B1 mutated	MDS	male	[85-90[
II-25	3900	8.9	146000	MDS SF3B1 mutated	MDS	female	[75-80[
II-26	21800	11.5	146000	MDS SF3B1 mutated	MDS	male	[70-75[

II-3	6600	7.9	333000	MDS SF3B1 mutated	MDS	male	[80-85[
II-4	3680	7.5	429000	MDS SF3B1 mutated	MDS	female	[70-75[
II-5	4930	9.9	220000	MDS SF3B1 mutated	MDS	male	[80-85[
II-6	47000	6.4	353000	MDS SF3B1 mutated	MDS	female	[65-70[
II-7	6800	8.7	284000	MDS SF3B1 mutated	MDS	male	[75-80[
II-8	5800	9.7	305000	MDS SF3B1 mutated	MDS	female	[70-75[
II-9	3000	9.2	109000	MDS SF3B1 mutated	MDS	male	[80-85[
III-1	4500	11.7	279000	MDS SF3B1 mutated	MDS	male	[85-90[
III-10	9800	10.2	433000	MDS SF3B1 mutated	MDS	male	[75-80[
III-11	6900	8.2	376000	MDS SF3B1 mutated	MDS	male	[75-80[
III-12	2600	8.2	304000	MDS SF3B1 mutated	MDS	female	[80-85[
III-13	NA	NA	NA	MDS SF3B1 mutated	MDS	male	[60-65[
III-14	7500	11	501000	MDS SF3B1 mutated	MDS	female	[80-85[
III-15	11800	11.7	780000	MDS SF3B1 mutated	MDS	male	[75-80[
III-16	6880	19.6	267000	MDS SF3B1 mutated	MDS	male	[65-70[
III-17	5500	10.3	337000	MDS SF3B1 mutated	MDS	male	[70-75[
III-18	1800	9.3	123000	MDS SF3B1 mutated	MDS	female	[80-85[
III-19	5600	10.7	275000	MDS SF3B1 mutated	MDS	female	[70-75[
III-2	NA	NA	NA	MDS SF3B1 mutated	MDS	male	[75-80[
III-20	2500	13	63000	MDS SF3B1 mutated	MDS	male	[40-45[
III-21	6400	10.7	331000	MDS SF3B1 mutated	MDS	female	[60-65[
III-22	NA	NA	NA	MDS SF3B1 mutated	MDS	male	[75-80[
III-23	4300	9.9	309000	MDS SF3B1 mutated	MDS	male	[75-80[
III-24	5700	9	207000	MDS SF3B1 mutated	MDS	male	[70-75[
III-25	6500	9.3	272000	MDS SF3B1 mutated	MDS	female	[55-60[
III-26	7800	9.6	296000	MDS SF3B1 mutated	MDS	male	[75-80[
III-27	5800	8.5	662000	MDS SF3B1 mutated	MDS	male	[85-90[
III-28	7600	9.9	332000	MDS SF3B1 mutated	MDS	female	[70-75[
III-29	4800	9.8	372000	MDS SF3B1 mutated	MDS	female	[65-70[
III-3	4600	10.9	299000	MDS SF3B1 mutated	MDS	male	[75-80[
III-30	2600	10.5	180000	MDS SF3B1 mutated	MDS	male	[70-75[
III-4	6500	8.6	258000	MDS SF3B1 mutated	MDS	male	[75-80[
III-5	8500	8.3	518000	MDS SF3B1 mutated	MDS	male	[65-70[
III-6	6300	8.7	572000	MDS SF3B1 mutated	MDS	male	[80-85[
III-7	6500	9.5	314000	MDS SF3B1 mutated	MDS	female	[75-80[
III-8	6700	10.3	284000	MDS SF3B1 mutated	MDS	male	[55-60[
III-9	4000	8.4	172000	MDS SF3B1 mutated	MDS	male	[85-90[
IV-1	6060	11.2	470000	MDS SF3B1 mutated	MDS	male	[55-60[
IV-10	5200	8.5	219000	MDS SF3B1 mutated	MDS	female	[75-80[
IV-11	7470	9.3	24000	MDS SF3B1 mutated	MDS	female	[85-90[
IV-12	5020	8.8	334000	MDS SF3B1 mutated	MDS	male	[75-80[
IV-13	3000	8.6	214000	MDS SF3B1 mutated	MDS	male	[70-75[
IV-14	6200	12.2	239000	MDS SF3B1 mutated	MDS	male	[65-70[
IV-15	7400	11	320000	MDS SF3B1 mutated	MDS	female	[60-65[

IV-16	9400	4.7	196000	MDS SF3B1 mutated	MDS	male	[85-90[
IV-17	3700	9.3	279000	MDS SF3B1 mutated	MDS	female	[70-75[
IV-18	5160	9.3	319000	MDS SF3B1 mutated	MDS	male	[70-75[
IV-19	9690	9.6	339000	MDS SF3B1 mutated	MDS	female	[70-75[
IV-2	9900	8.3	14000	MDS SF3B1 mutated	MDS	female	[80-85[
IV-20	NA	NA	NA	MDS SF3B1 mutated	MDS	male	[55-60[
IV-21	5700	9.4	399000	MDS SF3B1 mutated	MDS	female	[75-80[
IV-22	7600	11.3	173000	MDS SF3B1 mutated	MDS	male	[70-75[
IV-23	6700	8.3	382000	MDS SF3B1 mutated	MDS	male	[85-90[
IV-24	7120	9.8	163000	MDS SF3B1 mutated	MDS	male	[75-80[
IV-25	11000	8.2	463000	MDS SF3B1 mutated	MDS	male	[75-80[
IV-26	10500	10.8	233000	MDS SF3B1 mutated	MDS	male	[65-70[
IV-27	NA	NA	NA	MDS SF3B1 mutated	MDS	male	[80-85[
IV-28	5000	11.4	359000	MDS SF3B1 mutated	MDS	female	[75-80[
IV-29	3400	7.9	187000	MDS SF3B1 mutated	MDS	male	[70-75[
IV-3	8690	10.7	207000	MDS SF3B1 mutated	MDS	male	[75-80[
IV-30	8810	10.1	31700	MDS SF3B1 mutated	MDS	male	[70-75[
IV-4	3990	12.5	150000	MDS SF3B1 mutated	MDS	male	[70-75[
IV-5	6100	8.8	198000	MDS SF3B1 mutated	MDS	male	[80-85[
IV-6	NA	NA	NA	MDS SF3B1 mutated	MDS	female	[80-85[
IV-7	3000	10.8	282000	MDS SF3B1 mutated	MDS	male	[75-80[
IV-8	NA	NA	NA	MDS SF3B1 mutated	MDS	male	[85-90[
IV-9	NA	NA	NA	MDS SF3B1 mutated	MDS	male	[55-60[
IX-1	5700	8	257000	Iron deficiency anemia	anemia	female	[75-80[
IX-2	13240	6.9	805000	Iron deficiency anemia	anemia	female	[60-65[
IX-3	3030	10.5	8000	MDS U2AF1 mutated	MDS	male	[75-80[
IX-4	4200	12.6	104000	MDS SRSF2 mutated	MDS	female	[65-70[
IX-5	9800	8.6	313000	MDS U2AF1 mutated	MDS	male	[85-90[
IX-6	2900	5.4	80000	Megaloblastic anemia	anemia	male	[40-45[
V-1	5700	10	310000	MDS SF3B1 mutated	MDS	male	[70-75[
V-10	6600	11.5	231000	MDS SF3B1 mutated	MDS	male	[70-75[
V-2	3800	8.9	412000	MDS SF3B1 mutated	MDS	female	[75-80[
V-3	NA	NA	NA	MDS SF3B1 mutated	MDS	male	[70-75[
V-4	4660	10.6	164000	MDS SF3B1 mutated	MDS	female	[75-80[
V-5	4300	8.8	279000	MDS SF3B1 mutated	MDS	male	[70-75[
V-6	1630	10.7	54000	MDS SF3B1 mutated	MDS	male	[60-65[
V-7	9200	8.9	554000	MDS SF3B1 mutated	MDS	male	[70-75[
V-8	7200	10.5	255000	MDS SF3B1 mutated	MDS	male	[65-70[
V-9	4600	7.5	691000	MDS SF3B1 mutated	MDS	female	[75-80[
VI-1	NA	NA	NA	MDS SRSF2 mutated	MDS	male	[60-65[
VI-10	2700	10.1	25000	MDS U2AF1 mutated	MDS	male	[80-85[
VI-11	5810	12.3	103000	MDS U2AF1 mutated	MDS	male	[70-75[
VI-12	5970	7.3	134000	MDS U2AF1 mutated	MDS	male	[80-85[
VI-13	3600	13.8	163000	MDS SRSF2 mutated	MDS	male	[55-60[

VI-2	1600	6.4	74000	MDS SRSF2 mutated	MDS	male	[70-75[
VI-3	7100	11.6	38000	MDS RUNX1 mutated	MDS	female	[75-80[
VI-4	7600	8.4	164000	MDS RUNX1 mutated	MDS	female	[65-70[
VI-5	NA	NA	NA	MDS U2AF1 mutated	MDS	male	[75-80[
VI-6	1470	10.5	346000	MDS SRSF2 mutated	MDS	male	[80-85[
VI-7	1840	8.8	115000	MDS SRSF2 mutated	MDS	female	[65-70[
VI-8	6700	8.6	73000	MDS U2AF1 mutated	MDS	male	[80-85[
VI-9	1370	10	213000	MDS U2AF1 mutated	MDS	male	[70-75[
VII-1	5500	9.1	105000	MDS SRSF2 mutated	MDS	male	[75-80[
VII-10	9100	10.2	732000	Iron deficiency anemia	anemia	female	[75-80[
VII-11	2200	8.6	26000	MDS SRSF2 mutated	MDS	male	[75-80[
VII-12	4500	12.8	226000	MDS RUNX1 mutated	MDS	male	[70-75[
VII-13	2100	8.9	51000	MDS U2AF1 mutated	MDS	male	[50-55[
VII-14	8200	9.6	401000	MDS U2AF1 mutated	MDS	male	[65-70[
VII-15	NA	NA	NA	Iron deficiency anemia	anemia	female	[30-35[
VII-16	5920	11.3	318000	Megaloblastic anemia	anemia	female	[50-55[
VII-17	1500	6.3	54000	Megaloblastic anemia	anemia	male	[15-20[
VII-18	2400	11	137000	MDS SRSF2 mutated	MDS	female	[75-80[
VII-19	2600	14.2	89000	MDS SRSF2 mutated	MDS	male	[55-60[
VII-2	2590	9.4	98000	Megaloblastic anemia	anemia	female	[30-35[
VII-20	9520	10.9	25000	MDS SRSF2 mutated	MDS	female	[85-90[
VII-21	NA	NA	NA	MDS SRSF2 mutated	MDS	male	[80-85[
VII-22	4800	6.6	156600	Megaloblastic anemia	anemia	female	[50-55[
VII-23	2000	8.4	77000	Megaloblastic anemia	anemia	female	[75-80[
VII-24	2440	13.6	60000	MDS U2AF1 mutated	MDS	male	[60-65[
VII-25	1500	9.5	80000	MDS SRSF2 mutated	MDS	male	[70-75[
VII-26	4600	4.9	363000	MDS U2AF1 mutated	MDS	male	[50-55[
VII-27	1820	11.3	118000	MDS SRSF2 mutated	MDS	male	[60-65[
VII-28	2600	10.4	64000	MDS U2AF1 mutated	MDS	female	[80-85[
VII-29	5000	9	114000	MDS U2AF1 mutated	MDS	male	[80-85[
VII-3	4200	6.7	254000	Megaloblastic anemia	anemia	male	[40-45[
VII-30	2500	7.8	27000	MDS RUNX1 mutated	MDS	male	[65-70[
VII-4	3200	8.5	50000	MDS SRSF2 mutated	MDS	male	[75-80[
VII-5	3200	6.4	37000	Megaloblastic anemia	anemia	male	[55-60[
VII-6	10200	11.4	36000	MDS RUNX1 mutated	MDS	female	[65-70[
VII-7	2600	6.8	67000	Megaloblastic anemia	anemia	female	[50-55[
VII-8	2900	12.6	122000	MDS SRSF2 mutated	MDS	male	[75-80[
VII-9	2300	10.1	107000	MDS U2AF1 mutated	MDS	male	[75-80[
VIII-1	3950	8.4	17000	MDS RUNX1 mutated	MDS	male	[85-90[
VIII-10	5000	NA	NA	Megaloblastic anemia	anemia	female	[55-60[
VIII-11	2300	4.1	57000	Megaloblastic anemia	anemia	male	[65-70[
VIII-12	2240	8.9	41000	MDS U2AF1 mutated	MDS	male	[80-85[
VIII-13	7500	10.9	412000	Iron deficiency anemia	anemia	female	[20-25[
VIII-14	11000	9.2	330000	MDS U2AF1 mutated	MDS	male	[75-80[

VIII-15	2600	12.7	95000	MDS SRSF2 mutated	MDS	male	[75-80[
VIII-16	1910	6.6	49000	MDS RUNX1 mutated	MDS	male	[80-85[
VIII-17	1700	12.3	58000	MDS SRSF2 mutated	MDS	female	[65-70[
VIII-18	3870	12.8	144000	MDS U2AF1 mutated	MDS	female	[70-75[
VIII-19	4600	NA	11000	MDS RUNX1 mutated	MDS	male	[65-70[
VIII-2	17500	14.6	235000	Megaloblastic anemia	anemia	n.k	[60-65[
VIII-20	5600	8	164000	MDS RUNX1 mutated	MDS	female	[60-65[
VIII-21	2100	8.4	59000	MDS U2AF1 mutated	MDS	male	[80-85[
VIII-22	4300	8.6	292000	Iron deficiency anemia	anemia	female	[20-25[
VIII-23	5700	10	443000	Iron deficiency anemia	anemia	female	[40-45[
VIII-24	9180	9.3	40000	MDS SRSF2 mutated	MDS	male	[65-70[
VIII-3	3600	8.9	191000	MDS RUNX1 mutated	MDS	male	[80-85[
VIII-4	2700	10.5	82000	MDS U2AF1 mutated	MDS	female	[80-85[
VIII-5	6600	9.6	391000	Iron deficiency anemia	anemia	female	[35-40[
VIII-6	4800	7	127000	MDS U2AF1 mutated	MDS	male	[75-80[
VIII-7	6500	8.4	304000	Megaloblastic anemia	anemia	female	[50-55[
VIII-8	3900	6.8	129000	Megaloblastic anemia	anemia	female	[80-85[
VIII-9	NA	NA	NA	Megaloblastic anemia	anemia	female	[35-40[
X-1	4710	9.3	57000	MDS SRSF2 mutated	MDS	female	[85-90[
X-10	NA	NA	NA	MDS U2AF1 mutated	MDS	male	[75-80[
X-11	3220	6.2	47000	Megaloblastic anemia	anemia	male	[65-70[
X-12	2700	9	72000	MDS SRSF2 mutated	MDS	male	[50-55[
X-13	4000	8.1	168000	Megaloblastic anemia	anemia	male	[50-55[
X-14	2900	10	399000	MDS SRSF2 mutated	MDS	male	[75-80[
X-15	NA	NA	NA	Megaloblastic anemia	anemia	male	[25-30[
X-16	5400	10	49000	MDS SRSF2 mutated	MDS	female	[75-80[
X-2	5000	8.5	64000	Megaloblastic anemia	anemia	male	[55-60[
X-3	5800	10.8	306000	MDS U2AF1 mutated	MDS	female	[70-75[
X-4	10700	11.2	54000	Iron deficiency anemia	anemia	female	[40-45[
X-5	3300	14	100000	MDS SRSF2 mutated	MDS	male	[75-80[
X-6	3700	9.3	138000	Megaloblastic anemia	anemia	male	[15-20[
X-7	6900	8.9	349000	Iron deficiency anemia	anemia	female	[50-55[
X-8	NA	NA	NA	Normal finding	Normal	female	[75-80[
X-9	2500	9.2	95000	MDS SRSF2 mutated	MDS	male	[80-85[
XI-1	5100	9	172000	MDS SF3B1 mutated	MDS	female	[65-70[
XI-10	NA	NA	NA	MDS SF3B1 mutated	MDS	male	[70-75[
XI-11	5300	10.3	279000	MDS SF3B1 mutated	MDS	male	[75-80[
XI-12	4200	10.7	324000	MDS SF3B1 mutated	MDS	female	[75-80[
XI-13	5340	9.7	422000	MDS SF3B1 mutated	MDS	male	[65-70[
XI-14	6630	10.6	415000	MDS SF3B1 mutated	MDS	female	[65-70[
XI-15	2600	7.4	190000	MDS SF3B1 mutated	MDS	male	[80-85[
XI-16	6600	10.6	313000	MDS SF3B1 mutated	MDS	female	[75-80[
XI-2	3500	10.3	255000	MDS SF3B1 mutated	MDS	female	[70-75[
XI-3	1200	4.9	311000	MDS SF3B1 mutated	MDS	male	[40-45[

XI-4	5200	10.7	289000	MDS SF3B1 mutated	MDS	female	[65-70[
XI-5	5800	10.8	195000	MDS SF3B1 mutated	MDS	male	[70-75[
XI-6	4300	10.6	213000	MDS SF3B1 mutated	MDS	male	[70-75[
XI-7	4000	8.2	290000	MDS SF3B1 mutated	MDS	female	[80-85[
XI-8	NA	NA	NA	MDS SF3B1 mutated	MDS	female	[65-70[
XI-9	5270	8.7	304000	MDS SF3B1 mutated	MDS	male	[75-80[
XII-1	6500	9.8	243000	MDS SF3B1 mutated	MDS	male	[75-80[
XII-10	4140	8.6	221000	MDS SF3B1 mutated	MDS	female	[50-55[
XII-11	2900	7.5	287000	MDS SF3B1 mutated	MDS	female	[60-65[
XII-12	5400	8.9	167000	MDS SF3B1 mutated	MDS	male	[85-90[
XII-13	7300	8.7	264000	MDS SF3B1 mutated	MDS	female	[80-85[
XII-14	2240	NA	NA	MDS SF3B1 mutated	MDS	male	[80-85[
XII-15	6040	NA	NA	MDS SF3B1 mutated	MDS	male	[75-80[
XII-16	6100	8.6	258000	MDS SF3B1 mutated	MDS	female	[65-70[
XII-17	NA	NA	NA	MDS SF3B1 mutated	MDS	male	[80-85[
XII-18	7600	10.4	444000	MDS SF3B1 mutated	MDS	male	[75-80[
XII-2	4100	10	311000	MDS SF3B1 mutated	MDS	male	[80-85[
XII-20	1600	7.6	170000	MDS SF3B1 mutated	MDS	male	[75-80[
XII-3	3900	12.4	68000	MDS SF3B1 mutated	MDS	male	[75-80[
XII-4	3200	10	315000	MDS SF3B1 mutated	MDS	male	[60-65[
XII-5	5400	9.3	199000	MDS SF3B1 mutated	MDS	female	[80-85[
XII-6	5200	11.2	361000	MDS SF3B1 mutated	MDS	female	[65-70[
XII-7	3400	9.9	159000	MDS SF3B1 mutated	MDS	male	[55-60[
XII-8	5600	9.6	290000	MDS SF3B1 mutated	MDS	female	[75-80[
XII-9	NA	NA	NA	MDS SF3B1 mutated	MDS	male	[70-75[
XIII-1	NA	NA	NA	MDS RUNX1 mutated	MDS	male	[65-70[
XIII-10	6800	12.8	62000	MDS U2AF1 mutated	MDS	male	[80-85[
XIII-11	2500	10.7	163000	MDS U2AF1 mutated	MDS	female	[70-75[
XIII-12	3700	12.7	89000	MDS SRSF2 mutated	MDS	male	[80-85[
XIII-13	NA	NA	NA	Normal finding	Normal	male	[35-40[
XIII-14	7500	13.1	10000	MDS RUNX1 mutated	MDS	male	[75-80[
XIII-15	4100	11.9	232000	Normal finding	Normal	female	[35-40[
XIII-16	5800	16.1	195000	Normal finding	Normal	female	[80-85[
XIII-17	6000	13.5	244000	Normal finding	Normal	male	[70-75[
XIII-18	5900	13.8	229000	Normal finding	Normal	female	[40-45[
XIII-19	6300	13.3	406000	Normal finding	Normal	female	[40-45[
XIII-2	6700	14.2	372000	Normal finding	Normal	female	[45-50[
XIII-20	8200	15	229000	Normal finding	Normal	female	[50-55[
XIII-21	NA	NA	NA	Normal finding	Normal	female	[60-65[
XIII-22	NA	NA	NA	Normal finding	Normal	female	[50-55[
XIII-23	5700	17.1	278000	Normal finding	Normal	male	[45-50[
XIII-24	8100	16.8	240000	Normal finding	Normal	female	[70-75[
XIII-25	NA	NA	NA	Normal finding	Normal	female	[40-45[
XIII-26	8100	13.6	430000	Normal finding	Normal	female	[40-45[

XIII-27	5800	12.6	282000	Normal finding	Normal	female	[30-35[
XIII-28	8700	13.5	302000	Normal finding	Normal	female	[25-30[
XIII-29	5100	14.8	220000	Normal finding	Normal	female	[40-45[
XIII-3	3900	10.9	67000	MDS SRSF2 mutated	MDS	male	[75-80[
XIII-30	NA	NA	NA	Normal finding	Normal	male	[65-70[
XIII-4	5800	13.1	255000	Normal finding	Normal	female	[25-30[
XIII-5	NA	NA	NA	MDS RUNX1 mutated	MDS	male	[60-65[
XIII-6	3900	14.6	112000	MDS RUNX1 mutated	MDS	male	[75-80[
XIII-7	3900	8.4	46000	MDS RUNX1 mutated	MDS	female	[60-65[
XIII-8	1660	9.3	44000	MDS RUNX1 mutated	MDS	male	[75-80[
XIII-9	3800	9.9	38000	MDS RUNX1 mutated	MDS	male	[60-65[
XIV-1	7100	14.5	232000	Normal finding	Normal	male	[45-50[
XIV-2	NA	NA	NA	Normal finding	Normal	female	[50-55[
XIV-3	6300	12.5	228000	Normal finding	Normal	male	[65-70[
XV-1	8500	9.9	240000	MDS SRSF2 mutated	MDS	male	[65-70[
XV-10	12280	9.6	563000	Iron deficiency anemia	anemia	male	[55-60[
XV-11	2740	6.2	38000	Megaloblastic anemia	anemia	male	[55-60[
XV-12	3800	5	216000	Megaloblastic anemia	anemia	female	[15-20[
XV-13	4380	9.7	29000	Megaloblastic anemia	anemia	female	[40-45[
XV-14	2850	10.5	88000	Megaloblastic anemia	anemia	female	[80-85[
XV-15	2000	7.1	176000	Megaloblastic anemia	anemia	female	[55-60[
XV-16	4890	10	103000	MDS SRSF2 mutated	MDS	male	[75-80[
XV-17	2050	5.7	44000	Megaloblastic anemia	anemia	female	[40-45[
XV-18	1670	7	111000	Megaloblastic anemia	anemia	male	[60-65[
XV-19	2200	9.5	15000	Megaloblastic anemia	anemia	male	[70-75[
XV-2	NA	NA	NA	Megaloblastic anemia	anemia	female	[75-80[
XV-20	2910	8.1	119000	Megaloblastic anemia	anemia	male	[65-70[
XV-21	2870	8	167000	Megaloblastic anemia	anemia	female	[45-50[
XV-22	2600	5.2	74000	Megaloblastic anemia	anemia	female	[65-70[
XV-23	5500	7	418000	Iron deficiency anemia	anemia	female	[15-20[
XV-24	6340	9.3	361000	Iron deficiency anemia	anemia	female	[75-80[
XV-25	6200	8.5	292000	Iron deficiency anemia	anemia	female	[75-80[
XV-26	6300	7.9	86000	Megaloblastic anemia	anemia	female	[70-75[
XV-27	1380	6.8	38000	Megaloblastic anemia	anemia	male	[40-45[
XV-28	6900	8.4	498000	Iron deficiency anemia	anemia	female	[80-85[
XV-29	3900	8	329000	Iron deficiency anemia	anemia	female	[20-25[
XV-3	3580	9.6	245000	Megaloblastic anemia	anemia	male	[65-70[
XV-30	6900	8.8	190000	Iron deficiency anemia	anemia	female	[30-35[
XV-4	7350	7.5	164000	Megaloblastic anemia	anemia	female	[70-75[
XV-5	3100	14.4	84000	MDS SRSF2 mutated	MDS	male	[65-70[
XV-6	4100	4.2	189000	Megaloblastic anemia	anemia	female	[60-65[
XV-7	1400	14.4	127000	MDS SRSF2 mutated	MDS	male	[60-65[
XV-8	3400	2.2	6000	Megaloblastic anemia	anemia	female	[75-80[
XV-9	6000	9	180000	Iron deficiency anemia	anemia	female	[25-30[

XVI-1	5200	9.9	300000	Iron deficiency anemia	anemia	female	[15-20[
XVI-10	6100	8.9	126000	Megaloblastic anemia	anemia	male	[85-90[
XVI-11	5500	8.7	73000	Megaloblastic anemia	anemia	female	[80-85[
XVI-12	6100	11	285000	Iron deficiency anemia	anemia	female	[40-45[
XVI-13	1200	4.2	80000	Megaloblastic anemia	anemia	female	[45-50[
XVI-14	3000	8	123000	Iron deficiency anemia	anemia	male	[80-85[
XVI-15	4400	3.8	70000	Megaloblastic anemia	anemia	female	[20-25[
XVI-16	4700	11.8	92000	MDS SRSF2 mutated	MDS	male	[80-85[
XVI-17	9700	13.3	285000	Normal finding	Normal	female	[45-50[
XVI-18	NA	NA	NA	Normal finding	Normal	male	[55-60[
XVI-19	NA	NA	NA	Normal finding	Normal	female	[65-70[
XVI-2	5900	8.7	357000	Iron deficiency anemia	anemia	female	[50-55[
XVI-20	7900	13.7	306000	Normal finding	Normal	female	[40-45[
XVI-21	5900	10.5	188000	Normal finding	Normal	female	[30-35[
XVI-22	5700	18.5	92000	Normal finding	Normal	male	[75-80[
XVI-23	10400	11.6	32000	MDS U2AF1 mutated	MDS	female	[85-90[
XVI-24	4400	8.5	111000	MDS SRSF2 mutated	MDS	male	[75-80[
XVI-25	4400	12.1	225000	Normal finding	Normal	female	[75-80[
XVI-26	1400	9.5	26000	MDS RUNX1 mutated	MDS	male	[70-75[
XVI-27	1900	9.9	31500	MDS RUNX1 mutated	MDS	male	[85-90[
XVI-28	5100	11.7	256000	Normal finding	Normal	female	[50-55[
XVI-29	3300	12.4	48000	MDS RUNX1 mutated	MDS	male	[70-75[
XVI-3	7600	10.9	238000	Iron deficiency anemia	anemia	female	[55-60[
XVI-30	4700	12.4	156000	Normal finding	Normal	male	[40-45[
XVI-4	6200	9.4	326000	Iron deficiency anemia	anemia	female	[20-25[
XVI-5	6200	9.3	378000	Iron deficiency anemia	anemia	female	[70-75[
XVI-6	1600	4	26000	Megaloblastic anemia	anemia	female	[80-85[
XVI-7	2770	6.4	54000	Megaloblastic anemia	anemia	male	[45-50[
XVI-8	7700	9.9	589000	Iron deficiency anemia	anemia	female	[60-65[
XVI-9	8780	8.7	628000	Iron deficiency anemia	anemia	male	[45-50[
XVII-1	5400	9.2	694000	MDS U2AF1 mutated	MDS	male	[75-80[
XVII-10	6000	7.3	102000	MDS U2AF1 mutated	MDS	female	[80-85[
XVII-11	7300	16.2	145000	Normal finding	Normal	male	[35-40[
XVII-12	1700	7.8	53000	MDS RUNX1 mutated	MDS	male	[65-70[
XVII-13	1900	8.7	96000	MDS RUNX1 mutated	MDS	female	[75-80[
XVII-14	4930	19.8	208000	Normal finding	Normal	male	[55-60[
XVII-15	19370	8.9	218000	MDS SRSF2 mutated	MDS	male	[85-90[
XVII-16	NA	NA	NA	Normal finding	Normal	female	[15-20[
XVII-17	NA	NA	NA	MDS RUNX1 mutated	MDS	male	[70-75[
XVII-18	5300	7.7	29000	MDS RUNX1 mutated	MDS	male	[65-70[
XVII-19	2706	9.4	21900	MDS SRSF2 mutated	MDS	male	[70-75[
XVII-2	7500	NA	NA	Normal finding	Normal	male	[20-25[
XVII-20	3500	12.8	67000	MDS SRSF2 mutated	MDS	female	[65-70[
XVII-21	4600	NA	55000	MDS SRSF2 mutated	MDS	male	[75-80[

XVII-22	7400	9.6	6000	MDS RUNX1 mutated	MDS	male	[75-80[
XVII-23	9100	8.9	111000	MDS RUNX1 mutated	MDS	male	[80-85[
XVII-24	6300	10.3	177000	MDS SRSF2 mutated	MDS	male	[65-70[
XVII-25	3200	10.2	42000	MDS RUNX1 mutated	MDS	male	[65-70[
XVII-26	NA	NA	NA	MDS RUNX1 mutated	MDS	female	[70-75[
XVII-27	2200	11.1	216000	MDS RUNX1 mutated	MDS	female	[55-60[
XVII-28	6100	8.8	133000	MDS SRSF2 mutated	MDS	female	[55-60[
XVII-29	4200	10.2	34000	MDS RUNX1 mutated	MDS	female	[70-75[
XVII-3	6640	7.7	396000	MDS RUNX1 mutated	MDS	female	[70-75[
XVII-30	4120	9.7	97000	MDS RUNX1 mutated	MDS	male	[80-85[
XVII-4	2300	6.9	51000	MDS RUNX1 mutated	MDS	male	[60-65[
XVII-5	12790	8.8	58000	MDS RUNX1 mutated	MDS	male	[80-85[
XVII-6	3000	12	113000	MDS SRSF2 mutated	MDS	male	[60-65[
XVII-7	5800	14.1	220000	Normal finding	Normal	female	[35-40[
XVII-8	9400	14.6	399000	Normal finding	Normal	female	[60-65[
XVII-9	3100	11.4	183000	MDS SRSF2 mutated	MDS	male	[55-60[

Table S2: Blood counts and clinical classification of individuals in CUH2. NA = not available.

Slide ID	WBC/uL	Haemoglobin (g/dL)	Platelet/uL	Clinical classification	Clinical classification (simplified)
Iron-1	3700	8.7	214000	Iron deficiency anemia	anemia
Iron-10	3900	5.1	323000	Iron deficiency anemia	anemia
Iron-11	8500	7.5	290000	Iron deficiency anemia	anemia
Iron-12	7200	9.5	365000	Iron deficiency anemia	anemia
Iron-13	8800	9.3	191000	Iron deficiency anemia	anemia
Iron-14	5500	6.8	435000	Iron deficiency anemia	anemia
Iron-15	7100	4.9	298000	Iron deficiency anemia	anemia
Iron-2	11200	4.9	824000	Iron deficiency anemia	anemia
Iron-3	3900	7	266000	Iron deficiency anemia	anemia
Iron-4	4500	4.6	223000	Iron deficiency anemia	anemia
Iron-5	NA	NA	NA	Iron deficiency anemia	anemia
Iron-6	11000	7.8	586000	Iron deficiency anemia	anemia
Iron-7	8800	7.5	346000	Iron deficiency anemia	anemia
Iron-8	6400	9.1	603000	Iron deficiency anemia	anemia
Iron-9	NA	NA	NA	Iron deficiency anemia	anemia
Megaloblastic-1	NA	NA	NA	Megaloblastic anemia	anemia
Megaloblastic-2	NA	NA	NA	Megaloblastic anemia	anemia
Megaloblastic-3	2600	6.9	29000	Megaloblastic anemia	anemia
Megaloblastic-4	4900	2.7	35000	Megaloblastic anemia	anemia
Megaloblastic-5	2500	6.2	97000	Megaloblastic anemia	anemia
Megaloblastic-6	NA	NA	NA	Megaloblastic anemia	anemia
Megaloblastic-7	4900	2.7	35000	Megaloblastic anemia	anemia
Megaloblastic-8	NA	NA	NA	Megaloblastic anemia	anemia
Normal-N1	6900	13.3	216000	Normal finding	Normal
Normal-N10	7300	13.5	234000	Normal finding	Normal
Normal-N11	7600	11.9	191000	Normal finding	Normal
Normal-N2	8300	13.7	188000	Normal finding	Normal
Normal-N3	9200	12.4	224000	Normal finding	Normal
Normal-N4	6200	13.1	244000	Normal finding	Normal
Normal-N5	6300	12	172000	Normal finding	Normal
Normal-N6	7300	11.5	194000	Normal finding	Normal
Normal-N7	5400	15.4	189000	Normal finding	Normal
Normal-N8	8600	13.3	NA	Normal finding	Normal

Normal-N9	6700	13.4	228000	Normal finding	Normal
SF3B1-1	2600	7.8	102000	MDS SF3B1 mutated	MDS
SF3B1-11	6100	8.3	596000	MDS SF3B1 mutated	MDS
SF3B1-12	10100	11.2	861000	MDS SF3B1 mutated	MDS
SF3B1-13	NA	NA	NA	MDS SF3B1 mutated	MDS
SF3B1-14A	10400	9.2	660000	MDS SF3B1 mutated	MDS
SF3B1-14B	10400	9.2	660000	MDS SF3B1 mutated	MDS
SF3B1-2-RPT	6600	12.7	260000	MDS SF3B1 mutated	MDS
SF3B1-3	8100	8.6	55000	MDS SF3B1 mutated	MDS
SF3B1-4	5500	13.1	265000	MDS SF3B1 mutated	MDS
SF3B1-5-A	6500	9.6	294000	MDS SF3B1 mutated	MDS
SF3B1-5-B	6500	9.6	294000	MDS SF3B1 mutated	MDS
SF3B1-6-RPT	3700	10.2	302000	MDS SF3B1 mutated	MDS
SF3B1-7	2900	8.6	219000	MDS SF3B1 mutated	MDS
SF3B1-8-RPT	4000	9.6	792000	MDS SF3B1 mutated	MDS
SF3B1-9-1	4200	5	109000	MDS SF3B1 mutated	MDS
SF3B1-9-2A	2500	6.6	73000	MDS SF3B1 mutated	MDS
SF3B1-9-2B	2500	6.6	73000	MDS SF3B1 mutated	MDS
SRSF2-1	1770	11.9	293000	MDS SRSF2 mutated	MDS
SRSF2-10	2400	9.3	146000	MDS SRSF2 mutated	MDS
SRSF2-11	3200	8.5	35000	MDS SRSF2 mutated	MDS
SRSF2-12	3700	11.2	75000	MDS SRSF2 mutated	MDS
SRSF2-13	2400	8.4	135000	MDS SRSF2 mutated	MDS
SRSF2-14	8200	11.5	72000	MDS SRSF2 mutated	MDS
SRSF2-15	NA	NA	NA	MDS SRSF2 mutated	MDS
SRSF2-2	NA	NA	NA	MDS SRSF2 mutated	MDS
SRSF2-3	2400	13.5	151000	MDS SRSF2 mutated	MDS
SRSF2-4	2000	10.3	100000	MDS SRSF2 mutated	MDS
SRSF2-5	2300	14.1	204000	MDS SRSF2 mutated	MDS
SRSF2-6	3950	6.7	129000	MDS SRSF2 mutated	MDS
SRSF2-7	1600	12.9	130000	MDS SRSF2 mutated	MDS
SRSF2-8	18800	7.9	159000	MDS SRSF2 mutated	MDS
SRSF2-9	2900	13.2	127000	MDS SRSF2 mutated	MDS

Table S3: Augmentations used to train U-Net.

Augmentation	Explanation	Probability	Parameters
Elastic transform	A grid is randomly distorted using random gaussian noise leading to slight morphological changes in the cells	30%	$\sigma = 10$, no affine transformation
Gaussian blur	The image is blurred randomly using a convolution with side $2 \times$	0.1%	$\mu = 0, \sigma = 0.005, 1$
Random brightness (add.)	A random value within range is added to all the channels in the image	75%	$range = [-0.125, 0.12$
Random saturation (mult.)	The saturation (colourfulness of the image) is change by multiplying it by a random value within range	75%	$range = [0.7, 1.3]$
Random hue (add.)	The contrast (changes within the colour sphere, changing the colour of the image) is change by adding to it a random value within range	75%	$range = [-0.1, 0.1]$
Random contrast (mult.)	The contrast (the brightness range) is change by multiplying it by a random value within range	75%	$range = [0.7, 1.3]$
Salt and pepper noise	Randomly taking pixels and setting them to be 0 (minimum, black) or 1 (maximum, white)	1%	
Gaussian noise (add.)	Adding noise to all channels from a normal distribution centered in 0 and with standard deviation	100%	$\sigma = 0.05$

Table S4 - Mean number of detected cells and cellular density across conditions and cohorts. Cellular density was calculated as the ratio between number of detected cells and number of tiles predicted as being of good quality. For the Mean number of cells we provide the range (minimum and maximum values) in brackets.

Cell type	Dataset	Mean number of cells			Cellular density		
		Controls	MDS	Anemia	Controls	MDS	Anemia
RBC	MLLC	13426 [243,48310]	29326 [70,133916]	27334 [598,75239]	2.39	4.31	3.83
	CUH1	23725 [4813,64404]			3.55		
	CUH2	6314 [1826,18833]	24584 [2493,90379]	36384 [828,108979]	1.68	7.35	8.90
WBC	MLLC	1093 [187,2169]	1061 [80,5990]	1202 [319,4104]	0.22	0.19	0.20
	CUH1	4301 [311,36551]			0.74		
	CUH2	1054 [737,1694]	531 [50,1813]	793 [13,1844]	0.28	0.19	0.23

Table S5: Features used for morphometric characterisation.

Feature (count)	Description	Nuclear (count)
Area (1)	Area of detected object	X (1)
Perimeter (1)	Perimeter of detected object	X (1)
Eccentricity (1)	Ratio between major and minor axes	X (1)
Circle variance (1)	Measure of the difference between the contour and a circle fitted to the contour	X (1)
Ellipse variance (1)	Measure of the difference between the contour and an ellipse fitted to the contour	X (1)
Convexity (1)	Ratio between the perimeter of the convex hull of the contour and of the contour	X (1)
Solidity (1)	Ratio between the area of the convex hull of the contour and of the contour	X (1)
Centroid distance function (CDF) mean, standard deviation, minimum and maximum (4)	Descriptors for the distribution of values in the function characterising the distance between the object center and its edges	
CDF noise-insensitive moments (3)	Different ratios between the first four normalised moments ($\mu_1, \mu_2, \mu_3, \mu_4$) of the CDF ($\frac{\sqrt{\square}}{\square}$, $\frac{\mu_3}{\mu_2^{1.5}}$ and $\frac{\mu_4}{\mu_2^{2}}$) [17]	
Curvature function mean, standard deviation, minimum and maximum (4)	Descriptors for the distribution of values in the function characterising the curvature along the contour of the object	
Invariant region moments (7)	The first seven region moments. A more detailed description can be found in [17]	
CDF Fourier reconstruction error (1)	The area under the curve for the reconstruction error of a Fourier transform of both CDF. In practice: 1) a Fourier transform is fitted to a set of values; 2) the Fourier transform is used to construct a curve of reconstruction errors and 3) the area under the curve for the reconstruction error curve is calculated	
Standard deviation, Fourier reconstruction error, minimum and maximum of the intensity profile along the major (4) and minor axis (4) (8 in total)	Descriptors for the distribution of values in the function characterising the intensity profile along the major and minor axis. In practice: 1) the intensity values along an axis are extracted and 2) the standard deviation, Fourier reconstruction error, minimum and maximum are calculated for these values	
Mass displacement for the red, green and blue channels and the average intensity (4)	Difference in the center of mass between each colour channel and the average intensity and a uniform prior over the region	X (4)
Textural descriptors based on the gray level co-occurrence matrix (GLCM) (4)	Contrast, energy, homogeneity and correlation for GLCM. More details in [17]	

Table S6: Average run time, number of cells and “good” quality tiles for Haemorasis.

Decile	Median number of RBC	Median number of WBC	Median time per slide (seconds)	Median number of “good” quality tiles
**1st**	2263	572.	2445	2318
**2nd**	8398	922	4393	3687
**3rd**	11079	886.	6886	5301
**4th**	16579	1238	10105	6262
**5th**	14308	869	14503	4871
**6th**	17119	1312	22805	5744
**7th**	21924	1172	34516	5982
**8th**	31191	1216	48484	6415
**9th**	46695	1299	67943	7717
**10th**	57226	2763	81335	8904

Table S7: Comparison of external validation AUC with different subsets of WBC and RBC. Morph: models using only morphology; Morph+BC: models using morphology and blood counts.

Task	All WBC + all RBC		200 WBC + 200 RBC		500 WBC + 500 RBC	
	Morph	Morph+BC	Morph	Morph+BC	Morph	Morph+BC
**Disease detection**	86%	93%	67%	93%	72%	92%
**Disease classification**	88%	93%	87%	93%	87%	92%
**MDS genetic subtyping**	76%	87%	79%	85%	79%	85%
**Anemia classification**	98%	100%	99%	100%	98%	100%

Supplementary references

1. Fawcett, T. An introduction to ROC analysis. *Pattern Recognit. Lett.* **27**, 861–874 (2006).
2. Yang, S. J. *et al.* Assessing microscope image focus quality with deep learning. *BMC Bioinformatics* **19**, 77 (2018).
3. Huang, G., Liu, Z., Van Der Maaten, L. & Weinberger, K. Q. Densely connected convolutional networks. in *Proceedings of the IEEE conference on computer vision and pattern recognition* 4700–4708 (2017).
4. Canny, J. A computational approach to edge detection. *IEEE Trans. Pattern Anal. Mach. Intell.* **8**, 679–698 (1986).
5. Chen, T. & Guestrin, C. XGBoost: A Scalable Tree Boosting System. *arXiv [cs.LG]* (2016).
6. Rashmi, K. V. & Gilad-Bachrach, R. DART: Dropouts meet Multiple Additive Regression Trees. *Proceedings of Machine Learning Research* (2015).
7. Abramson, N. Rouleaux formation. *Blood* **107**, 4205 (2006).
8. Mullins, M. *et al.* Cold agglutinin disease burden: a longitudinal analysis of anemia, medications, transfusions, and health care utilization. *Blood Adv* **1**, 839–848 (2017).
9. Falk, T. *et al.* U-Net: deep learning for cell counting, detection, and morphometry. *Nat. Methods* **16**, 67–70 (2018).
10. Kingma, D. P. & Ba, J. Adam: A Method for Stochastic Optimization. *arXiv [cs.LG]* (2014).
11. Tellez, D. *et al.* Quantifying the effects of data augmentation and stain color normalization in convolutional neural networks for computational pathology. *Med. Image Anal.* **58**, 101544 (2019).
12. Moshkov, N., Mathe, B., Kertesz-Farkas, A., Hollandi, R. & Horvath, P. Test-time augmentation for deep learning-based cell segmentation on microscopy images. *Sci. Rep.* (2020) doi:10.1038/s41598-020-61808-3.
13. Andrade, A. R. *et al.* Recent computational methods for white blood cell nuclei segmentation: A comparative study. *Comput. Methods Programs Biomed.* **173**, 1–14 (2019).
14. Buslaev, A. *et al.* Albumentations: Fast and Flexible Image Augmentations. *Information* **11**, 125 (2020).
15. Sommer, C., Straehle, C., Kothe, U. & Hamprecht, F. A. Ilastik: Interactive learning and segmentation toolkit. in *Proceedings - International Symposium on Biomedical Imaging* (2011). doi:10.1109/ISBI.2011.5872394.
16. Carpenter, A. E. *et al.* CellProfiler: Image analysis software for identifying and quantifying cell phenotypes. *Genome Biol.* (2006) doi:10.1186/gb-2006-7-10-r100.
17. Mingqiang, Y., Kidiyo, K. & Joseph, R. A Survey of Shape Feature Extraction Techniques. in *Pattern Recognition Techniques, Technology and Applications* (2008).
18. McInnes, L., Healy, J., Saul, N. & Großberger, L. UMAP: Uniform Manifold Approximation and Projection. *Journal of Open Source Software* (2018) doi:10.21105/joss.00861.
19. Bostock, M., Ogievetsky, V. & Heer, J. D^3: Data-Driven Documents. *IEEE Trans. Vis. Comput. Graph.* **17**, 2301–2309 (2011).
20. Valent, P. *et al.* Proposed minimal diagnostic criteria for myelodysplastic syndromes (MDS) and potential pre-MDS conditions. *Oncotarget* **8**, 73483–73500 (2017).

21. Amores, J. Multiple instance classification: Review, taxonomy and comparative study. *Artif. Intell.* **201**, 81–105 (2013).
22. Ruchte, M. & Grabocka, J. Efficient Multi-Objective Optimization for Deep Learning. *arXiv [cs.LG]* (2021).
23. Censor, Y. Pareto optimality in multiobjective problems. *Appl. Math. Optim.* **4**, 41–59 (1977).
24. Merkel, D. & Others. Docker: lightweight linux containers for consistent development and deployment. *Linux J.* **2014**, 2 (2014).
25. Goode, A., Gilbert, B., Harkes, J., Jukic, D. & Satyanarayanan, M. OpenSlide: A vendor-neutral software foundation for digital pathology. *J. Pathol. Inform.* **4**, 27 (2013).
26. Bankhead, P. *et al.* QuPath: Open source software for digital pathology image analysis. *Sci. Rep.* **7**, 16878 (2017).
27. Van Rossum & Drake. The python language reference. *Python software foundation*.
28. R Core Team. R: A Language and Environment for Statistical Computing. Preprint at https://www.R-project.org/ (2020).
29. Harris, C. R. *et al.* Array programming with NumPy. *Nature* **585**, 357–362 (2020).
30. Virtanen, P. *et al.* SciPy 1.0: fundamental algorithms for scientific computing in Python. *Nat. Methods* **17**, 261–272 (2020).
31. Murray, A. *et al. python-pillow/Pillow: 9.3.0*. (2022). doi:10.5281/zenodo.7263546.
32. van der Walt, S. *et al.* scikit-image: image processing in Python. *PeerJ* **2**, e453 (2014).
33. Bradski, G. The openCV library. *Dr. Dobb's Journal: Software Tools for the Professional Programmer* **25**, 120–123 (2000).
34. Pedregosa, F. *et al.* Scikit-learn: Machine Learning in Python. *J. Mach. Learn. Res.*
35. Abadi, M. *et al.* TensorFlow: A System for Large-Scale Machine Learning. in *12th USENIX Symposium on Operating Systems Design and Implementation (OSDI '16)* 265–284 (2016).
36. Paszke, A. *et al.* PyTorch: An imperative style, high-performance deep learning library. *Adv. Neural Inf. Process. Syst.* **32**, (2019).
37. Collette, A. & Others. h5py: HDF5 for Python. Preprint at (2019).
38. McKinney, W. Data Structures for Statistical Computing in Python. in *Proceedings of the 9th Python in Science Conference* (eds. van der Walt, S. & Millman, J.) (SciPy, 2010). doi:10.25080/majora-92bf1922-00a.
39. Mölder, F. *et al.* Sustainable data analysis with Snakemake. *F1000Res.* **10**, 33 (2021).
40. Wickham, H. The tidyverse. *R package ver*.
41. Jerome, A., Hastie, T., Tibshirani, R. & Simon, N. *Package ' glmnet '*. (2019).
42. Kuhn, M. caret: Classification and Regression Training. *Astrophysics Source Code Library* ascl:1505.003 Preprint at https://ui.adsabs.harvard.edu/abs/2015ascl.soft05003K (2015).
43. Yan, Y. MLmetrics: Machine learning evaluation metrics. *R package version*.
44. Robin, X. *et al.* pROC: an open-source package for R and S+ to analyze and compare ROC curves. *BMC Bioinformatics* **12**, 77 (2011).
45. Wickham, H. Data Analysis. in *ggplot2: Elegant Graphics for Data Analysis* (ed. Wickham, H.) 189–201 (Springer International Publishing, 2016).
46. Kassambara, A. ggpubr:'ggplot2' based publication ready plots. (2020).

47. Xiao, N. ggsci: scientific journal and sci-fi themed color palettes for ‘ggplot2’. *R package version*.

48. Venables, W. N. & Ripley, B. D. MASS: modern applied statistics with S. *R package version*.

49. Dinno, A. dunn. test: Dunn’s test of multiple comparisons using rank sums. *R package version*.

50. Konopka, T. Umap: uniform manifold approximation and projection. *R package version 0.2*.

51. Malcovati, L. *et al.* Diagnosis and treatment of primary myelodysplastic syndromes in adults: recommendations from the European LeukemiaNet. *Blood* **122**, 2943–2964 (2013).

52. Brück, O. E. *et al.* Machine Learning of Bone Marrow Histopathology Identifies Genetic and Clinical Determinants in Patients with MDS. *Blood Cancer Discov* **2**, 238–249 (2021).

53. Cui, R. *et al.* Clinical importance of SF3B1 mutations in Chinese with myelodysplastic syndromes with ring sideroblasts. *Leuk. Res.* **36**, 1428–1433 (2012).
