# Supplementary material for: Computational analysis of peripheral blood smears detects disease-associated cytomorphologies
Source: Nat Commun. 2023 Jul 20;14:4378. doi: 10.1038/s41467-023-39676-y (PMC10359268; doi:10.1038/s41467-023-39676-y)
Supplement: Supplementary file 3 — Reporting Summary [file 41467_2023_39676_MOESM3_ESM.pdf]

nature portfolio

Corresponding author(s): Moritz Gerstung
George S. Vassiliou

Last updated by author(s): Nov 3, 2022

Reporting Summary

Nature Portfolio wishes to improve the reproducibility of the work that we publish. This form provides structure for consistency and transparency in reporting. For further information on Nature Portfolio policies, see our Editorial Policies and the Editorial Policy Checklist.

Statistics

For all statistical analyses, confirm that the following items are present in the figure legend, table legend, main text, or Methods section.

n/a	Confirmed	
☐	☒	The exact sample size (*n*) for each experimental group/condition, given as a discrete number and unit of measurement
☒	☐	A statement on whether measurements were taken from distinct samples or whether the same sample was measured repeatedly
☐	☒	The statistical test(s) used AND whether they are one- or two-sided<br>*Only common tests should be described solely by name; describe more complex techniques in the Methods section.*
☐	☒	A description of all covariates tested
☐	☒	A description of any assumptions or corrections, such as tests of normality and adjustment for multiple comparisons
☐	☒	A full description of the statistical parameters including central tendency (e.g. means) or other basic estimates (e.g. regression coefficient) AND variation (e.g. standard deviation) or associated estimates of uncertainty (e.g. confidence intervals)
☐	☒	For null hypothesis testing, the test statistic (e.g. *F*, *t*, *r*) with confidence intervals, effect sizes, degrees of freedom and *P* value noted<br>*Give P values as exact values whenever suitable.*
☒	☐	For Bayesian analysis, information on the choice of priors and Markov chain Monte Carlo settings
☒	☐	For hierarchical and complex designs, identification of the appropriate level for tests and full reporting of outcomes
☐	☒	Estimates of effect sizes (e.g. Cohen's *d*, Pearson's *r*), indicating how they were calculated

Our web collection on statistics for biologists contains articles on many of the points above.

Software and code

Policy information about availability of computer code

Data collection	NDP.scan (version 3.2.12) was used to digitize peripheral blood slides with the Hammamatsu Nanozoomer 2 scanners. The Aperio AT2 DX scanner was used to digitize peripheral blood slides with the Scanscope (version 102.0.75) software.
Data analysis	We have made the Haemorasis pipeline available in https://github.com/josegcpa/haemorasis and as a Docker container in https://hub.docker.com/repository/docker/josegcpa/blood-cell-detection. Morphotype analysis (mil-comori) and the statistical analysis and plot generation code (analysis-plotting) are available at https://github.com/josegcpa/wbs-prediction. The code for the quality control network is available at https://github.com/josegcpa/quality-net. The code for the U-Net is available at https://github.com/josegcpa/u-net-tf2.<br><br>We used Python 3.6.8 with the following packages (versions are specified after a "==" sign): albumentations==1.1.0, h5py==2.10.0, numpy==1.19.2, opencv-contrib-python==4.4.0.44, opencv-python==4.4.0.44, opencv-python-headless==4.4.0.44, openslide-python==1.1.2, pandas==1.1.5, Pillow==7.2.0, scikit-image==0.17.2, scikit-learn==0.23.2, scipy==1.5.2, snakemake==6.10.0, tensorflow==2.0, torch==1.10.1, xgboost=1.3. We used R v3.6.1 with the following packages (versions are specified after a "==" sign): caret==6.0-83, cowplot==1.1.1, dunn.test==1.3.5, ggplot2==3.3.5, ggpubr==0.4.0,17 ggsci==2.9, glmnet==2.0-16, MASS==7.3-51.3, MLmetrics==1.1.1, pROC==1.18.0, tidyverse==1.3.1, umap==0.2.7.0.<br><br>QuPath==0.3 was also used.

For manuscripts utilizing custom algorithms or software that are central to the research but not yet described in published literature, software must be made available to editors and reviewers. We strongly encourage code deposition in a community repository (e.g. GitHub). See the Nature Portfolio guidelines for submitting code & software for further information.

Data

Policy information about availability of data

All manuscripts must include a data availability statement. This statement should provide the following information, where applicable:

- Accession codes, unique identifiers, or web links for publicly available datasets
- A description of any restrictions on data availability
- For clinical datasets or third party data, please ensure that the statement adheres to our policy

The digitized PBS image data generated in this study and used for training (MLL) have been deposited in the BioImage Archive database under accession code S-BIAD440 (https://www.ebi.ac.uk/biostudies/BioImages/studies/S-BIAD440). The annotated datasets for tile quality classification, white blood cell segmentation and red blood cell filtering are available in https://doi.org/10.6084/m9.figshare.19153760. The machine-learning model parameters are available at https://doi.org/10.6084/m9.figshare.19164209. The necessary data to run Morphotype analysis is available at https://doi.org/10.6084/m9.figshare.19372292. The output of the Morphotype analysis, as well as the expert annotated cells, and the data necessary for downstream analysis are available at https://doi.org/10.6084/m9.figshare.19369391 and https://doi.org/10.6084/m9.figshare.19371008, respectively. An online platform for morphotype visualization is available in https://https://josegcpa.github.io/haemorasis-umap and the data supporting it is available in https://github.com/josegcpa/json-haemorasis.

Human research participants

Policy information about studies involving human research participants and Sex and Gender in Research.

Reporting on sex and gender	We have focused on reporting sex rather than gender. Information on sex/gender was not collected for the CUH1 cohort. All Munich Leukemia Laboratory data provided for this investigation were reviewed and approved by Munich Leukemia Laboratory's internal institutional review board and follow the European Union's General Data Protection Regulation (GDPR). This research was conducted in line with the European Molecular Biology Laboratory's internal policy 53 (Internal Policy regarding the Use of Human Biological Material).
Population characteristics	The MLL cohort was composed of 203 male and 159 female individuals, with mean age 66.1 (362 individuals in total); 58 individuals were hematological normal controls, 131 had SF3B1-mutant MDS, 40 had SRSF2-mutant MDS, 34 had RUNX1-mutant MDS, 28 had U2AF1-mutant MDS (233 individuals with MDS), 27 had iron deficiency anemia and 44 had megaloblastic anemia (71 individuals with anemia). The CUH2 cohort was composed of 28 male and 34 female individuals (62 individuals in total, for 6 no information on sex was found), with mean age 58.3; 11 individuals were hematological normal controls, 19 had SF3B1-mutant MDS, 15 had SRSF2-mutant MDS (34 individuals with MDS), 15 had iron deficiency anemia and 8 had megaloblastic anemia (23 individuals with anemia).
Recruitment	This study was retrospective so no human participant recruitment was necessary.
Ethics oversight	MLL data was collected and digitized with individual informed consent for research purposes and the study was reviewed and approved by the Munich Leukemia Laboratory's internal institutional review board and follow the European Union's General Data Protection Regulation (GDPR). Regarding data from Addenbrooke, the study was approved by the National Health Service Health Research Authority and the Health and Care Research Wales (Research Ethics Committee reference: 23/PR/0578).

Note that full information on the approval of the study protocol must also be provided in the manuscript.

Field-specific reporting

Please select the one below that is the best fit for your research. If you are not sure, read the appropriate sections before making your selection.

[x] Life sciences [ ] Behavioural & social sciences [ ] Ecological, evolutionary & environmental sciences

For a reference copy of the document with all sections, see nature.com/documents/nr-reporting-summary-flat.pdf

Life sciences study design

All studies must disclose on these points even when the disclosure is negative.

Sample size	No sample size was determined a priori for either cohort (CUH1, CUH2, MLL); in any case we focused on getting as many cases as possible to ensure that we had large-enough numbers to both develop and test the here algorithms and models here presented.
Data exclusions	Inadequately prepared (excessive amount of stain) or scanned slides (the whole slide was blurred) were excluded from further analysis (11 slides excluded from the MLL cohort and 1 from the CUH2 cohort). 4 additional slides were excluded from CUH2 as they were repeated, keeping only one slide per individual.
Replication	We have externally validated our predictive performance results on an external cohort obtained from a different hospital and using a different slide scanner. After training our models (glmnet models and morphotype analysis models) on the training cohort (MLL), we tested all models - glmnet models, morphotype analysis models with all morphotypes and morphotype analysis models only with stable morphotypes - on the

	CUH2 cohort, our validation cohort. We show that, excluding the disease detection model using all morphotypes, all models are generalizable, i.e. the performance in the validation set is similar to that of the cross-validation in the training set.
Randomization	Randomization was performed during training with stratified cross-validation. During this process, individuals were randomly split into one of 5 non-overlapping subsets of the training data, the union of which yields the complete training dataset. The allocation of individuals to each fold was random within each one of the relevant classes (controls, SF3B1-positive MDS, SF3B1-negative MDS, megaloblastic anemia and iron deficiency anemia) such that the proportion of individuals with a given condition in each fold was as similar as possible to the proportion of individuals with that same condition in the training set.
Blinding	Data collection was performed in order to construct a cohort of individuals with a range of diseases - SF3B1-positive MDS, SF3B1-negative MDS, megaloblastic anemia and iron deficiency anemia - as well as controls, so the selection of patients was not blinded. Within each of these groups no relevant criteria was used to select patients.

Reporting for specific materials, systems and methods

We require information from authors about some types of materials, experimental systems and methods used in many studies. Here, indicate whether each material, system or method listed is relevant to your study. If you are not sure if a list item applies to your research, read the appropriate section before selecting a response.

Materials & experimental systems

n/a	Involved in the study
☒	☐ Antibodies
☒	☐ Eukaryotic cell lines
☒	☐ Palaeontology and archaeology
☒	☐ Animals and other organisms
☒	☐ Clinical data
☒	☐ Dual use research of concern

Methods

n/a	Involved in the study
☒	☐ ChIP-seq
☒	☐ Flow cytometry
☒	☐ MRI-based neuroimaging
